# Baseline data of parasite clearance in patients with falciparum malaria treated with an artemisinin derivative: an individual patient data meta-analysis

**DOI:** 10.1186/s12936-015-0874-1

**Published:** 2015-09-22

**Authors:** WWARN Parasite Clearance Study Group

**Affiliations:** WorldWide Antimalarial Resistance Network, Centre for Tropical Medicine and Global Health, Nuffield Department of Medicine Research Building, University of Oxford, Old Road Campus, Roosevelt Drive, Oxford, OX3 7FZ UK

**Keywords:** Malaria, Parasite clearance, Artemisinin resistance, Drug resistance, *Plasmodium falciparum*

## Abstract

**Background:**

Artemisinin resistance in *Plasmodium falciparum* manifests as slow parasite clearance but this measure is also influenced by host immunity, initial parasite biomass and partner drug efficacy. This study collated data from clinical trials of artemisinin derivatives in falciparum malaria with frequent parasite counts to provide reference parasite clearance estimates stratified by location, treatment and time, to examine host factors affecting parasite clearance, and to assess the relationships between parasite clearance and risk of recrudescence during follow-up.

**Methods:**

Data from 24 studies, conducted from 1996 to 2013, with frequent parasite counts were pooled. Parasite clearance half-life (PC_1/2_) was estimated using the WWARN Parasite Clearance Estimator. Random effects regression models accounting for study and site heterogeneity were used to explore factors affecting PC_1/2_ and risk of recrudescence within areas with reported delayed parasite clearance (western Cambodia, western Thailand after 2000, southern Vietnam, southern Myanmar) and in all other areas where parasite populations are artemisinin sensitive.

**Results:**

PC_1/2_ was estimated in 6975 patients, 3288 of whom also had treatment outcomes evaluate d during 28–63 days follow-up, with 93 (2.8 %) PCR-confirmed recrudescences. In areas with artemisinin-sensitive parasites, the median PC_1/2_ following three-day artesunate treatment (4 mg/kg/day) ranged from 1.8 to 3.0 h and the proportion of patients with PC_1/2_ >5 h from 0 to 10 %. Artesunate doses of 4 mg/kg/day decreased PC_1/2_ by 8.1 % (95 % CI 3.2–12.6) compared to 2 mg/kg/day, except in populations with delayed parasite clearance. PC_1/2_ was longer in children and in patients with fever or anaemia at enrolment. Long PC_1/2_ (HR = 2.91, 95 % CI 1.95–4.34 for twofold increase, p < 0.001) and high initial parasitaemia (HR = 2.23, 95 % CI 1.44–3.45 for tenfold increase, p < 0.001) were associated independently with an increased risk of recrudescence. In western Cambodia, the region with the highest prevalence of artemisinin resistance, there was no evidence for increasing PC_1/2_ since 2007.

**Conclusions:**

Several factors affect PC_1/2_. As substantial heterogeneity in parasite clearance exists between locations, early detection of artemisinin resistance requires reference PC_1/2_ data. Studies with frequent parasite count measurements to characterize PC_1/2_ should be encouraged. In western Cambodia, where PC_1/2_ values are longest, there is no evidence for recent emergence of higher levels of artemisinin resistance.

**Electronic supplementary material:**

The online version of this article (doi:10.1186/s12936-015-0874-1) contains supplementary material, which is available to authorized users.

## Background

Parasite clearance is a robust measure of the efficacy of anti-malarial drugs, which has been used particularly to measure the pharmacodynamic effects of artemisinin derivatives [1]. Initial studies conducted in patients with severe malaria employed frequent parasite counting to characterize clearance profiles, and these demonstrated that artemisinin derivatives cleared parasitaemia more rapidly than quinine [2]. More recently, frequent parasite counting has been used to characterize artemisinin susceptibility in vivo [3], and to validate molecular markers [4, 5] and in vitro assays for detection of artemisinin resistance [6]. Parasite clearance following artemisinin treatment is influenced by a number of factors other than parasite susceptibility, including host immunity, initial parasite biomass and partner drug efficacy. It is therefore essential to control for such potential confounding factors in order to identify temporal changes in parasite clearance resulting from reduced anti-malarial drug susceptibility.

The WorldWide Antimalarial Resistance Network (WWARN) Parasite Clearance Estimator (PCE) [7] was developed to automate and standardize analysis of frequent parasite count data. This tool is freely available online [8] and provides an automated report for each patient. The derived measure, parasite clearance half-life (PC_1/2_), generated by the PCE reflects the extent to which ring-stage parasites are killed and removed from the circulation, and is currently considered the most reliable measure of parasitological responses to treatment with artemisinin or its derivatives [5, 9–16]. This standardized approach to PC_1/2_ measurement allows comparison in space and time of artemisinin resistance, which manifests as a slow parasite clearance rate in patients. Within the WWARN framework, investigators who obtained frequent parasite count data have joined several study groups [17] to evaluate this metric. This pooled analysis presents reference parasite clearance estimates stratified by geographic location, treatment and study population, and explores the relationship between parasite clearance measures and the risk of recrudescent infection (treatment failure). The effects of different sampling strategies on clearance estimates have been published separately [18].

## Methods

### Data acquisition

Any study involving patients with uncomplicated falciparum malaria, treated with either artemisinin combination therapy (ACT) or oral artesunate monotherapy, in which peripheral parasitaemia was measured at least twice daily in the first 3 days after starting treatment, was eligible for inclusion in this pooled analysis. In addition, the minimum data required were enrolment date, patient age, drug treatment, study location and characteristics, and details of the parasite counting method. Studies with frequent parasite counts were identified using literature reviews and existing collaborations within WWARN. Principal investigators were subsequently approached to participate in this study group [19]. The datasets uploaded to the WWARN repository were standardized using the WWARN Data Management and Statistical Analysis Plans for clinical data [20] and pooled into a single database of quality-assured individual patient data.

Parasite inclusion criteria, counting methods and blood sampling schedules were different among studies; for a detailed description see Additional file 1: Table S1.

### Statistical analysis

#### Definitions

As a measure of transmission intensity, malaria endemicity estimates were obtained for study sites and year from the Malaria Atlas Project [21]. Anaemia was defined according to WHO guidelines [22], (i.e., haemoglobin concentration cut-offs for moderate anaemia were 10 g/dL in children <5 years of age and 11 g/dL in older patients, and for severe anaemia were 7 and 8 g/dL, respectively). For studies where haematocrit only was measured, the following relationship was used to estimate haemoglobin: haematocrit (%) = 5.62 + 2.60 × haemoglobin (g/dL) [23]. Nutritional status of children aged <5 years was assessed by the weight-for-age indicator using the *igrowup* package developed by the WHO [24].

#### Analysis of parasite counts

PC_1/2_ was estimated only for patients with sufficient parasite counts defined as sampling at least 12-h in the first 48 h (a maximum of a 16-h gap between any two measurements, as a 2-h window on each side was allowed) and at least 24-h sampling (maximum 28-h gap) after 48 h until parasite clearance [18]. The following deviations from this rule were accepted as they were deemed not to have substantial effects on the PC_1/2_ estimate [18]: sampling was not performed until parasite clearance but the last recorded parasitaemia was <100 or <1000 parasites/µL with at least five positive parasite counts available; a longer gap was observed between a set of measurements but there were at least two positive parasite counts directly after the gap, or a zero count was recorded after the gap and the last recorded parasitaemia before the gap was either <100 or <1000/µL and at least five positive parasite count measurements were available before the gap.

PC_1/2_ was calculated by the PCE [7] for each patient (variable called slope_half_life in the output files), based on the linear segment of the decline in the log-transformed parasitaemia-time profile. A lag-phase (an initial, flat part of the parasitaemia-time profile which precedes the log-linear decline) and a tail (a levelling out in the parasitaemia-time profile which follows the log-linear decline), if present, are identified by the PCE automatically.

Reliability of PC_1/2_ estimates was assessed by (a) the standard deviation of residuals from the final linear model used to estimate PC_1/2_; (b) the duration of the lag phase (as a long lag phase is very unlikely if an artemisinin derivative is given and absorption is adequate); (c) the number of positive parasite counts used in the estimation; (d) pseudo-R^2^ statistics; and, (e) the width of the 95 % confidence interval around the PC_1/2_.

Pseudo-R^2^ is a measure of goodness of fit of the final model and is provided by the PCE tool. Low values of pseudo-R^2^ indicate that the predicted values from the polynomial model are far from the measured parasitaemias. Pseudo-R^2^ is calculated from the fitted values of the final linear model used to estimate the PC_1/2_ (after exclusion of the lag phase and tail) and the observed log-parasitaemias, excluding zero counts.

#### Parasite clearance and clinical covariates

Factors affecting PC_1/2_ were investigated in the random effects regression model (to account for study site heterogeneity) with PC_1/2_ being modelled after log transformation. Separate analyses were performed in artemisinin-resistant and artemisinin-sensitive areas. The resistant areas were defined as locations in which delayed parasite clearance had been reported previously [3, 9–11, 14–16, 25–28] (i.e., western Cambodia, western Thailand after 2000, southern Vietnam, southern Myanmar), while the sensitive areas were defined as all other locations.

In studies which randomized treatment arms to 2 and 4 mg/kg/day artesunate doses, meta-analysis of the differences in mean log-transformed PC_1/2_ between treatment arms was performed using a fixed effects model using the inverse variance method. Heterogeneity was evaluated by *I*^2^ [29].

#### Analysis of treatment outcome

The risk of recrudescence was assessed by survival analysis using WHO definitions of therapeutic efficacy outcome [30]. Patients with no PCR results were excluded from the treatment outcome analysis. Cox regression model with random effects in the form of frailty parameters were used to adjust for study site effects [31]. The proportional hazard assumption was tested based on Schoenfeld residuals [32]. PC_1/2_, presence of a lag phase, duration of lag phase and presence of a tail were evaluated as possible predictors of outcome, together with all other baseline clinical and treatment characteristics.

Covariates for the final regression models (for treatment outcome and PC_1/2_) were selected on the basis of the likelihood ratio test and examination of residuals. Relationship between the independent variable and continuous covariates such as age and parasitaemia was examined using fractional polynomials. All statistical analyses were performed using Stata 13.0.

## Results

### Data summary

**Fig. 1:**
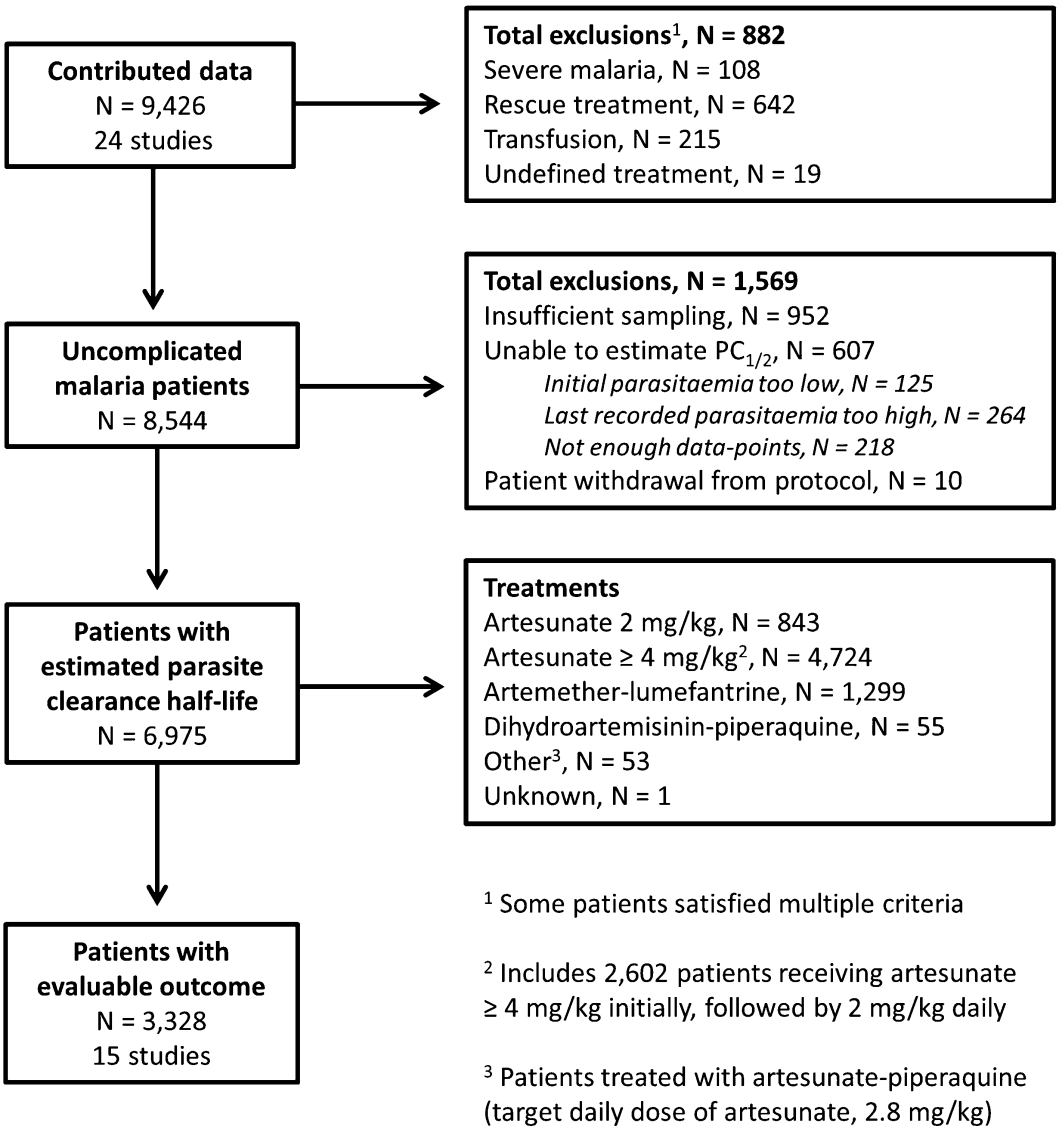
Study profile

**Fig. 2:**
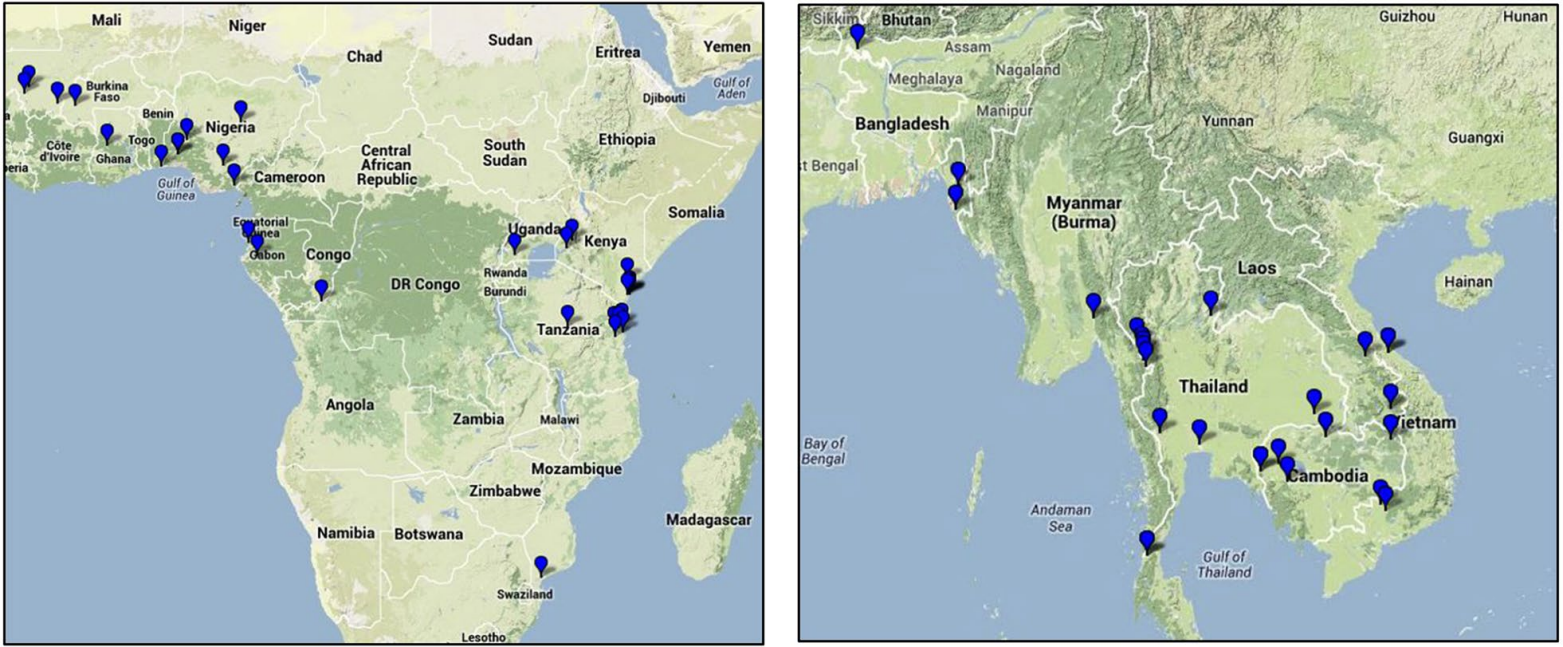
Map of study sites included in the parasite clearance data analysis

Data from 9318 patients enrolled from 1996 to 2013 in 24 studies [3, 9–15, 26, 33–43] (Additional file 2: Table S2; Fig. 1) conducted at 61 study sites in 46 distinct locations (Fig. 2) in 18 countries (Bangladesh, Benin, Burkina Faso, Cambodia, Democratic Republic of Congo, Gabon, Ghana, India, Kenya, Laos, Mali, Mozambique, Myanmar, Nigeria, Tanzania, Thailand, Uganda, Vietnam), were available for analysis.

Among hyperparasitaemic patients (Study ID 1), 882 of 3393 (26 %) patients were excluded from analysis because of one or more of the following: severe malaria (n = 108), slow parasite clearance and administration of rescue treatment with intravenous or intramuscular artesunate (n = 642), blood transfusion before clearance of parasites (n = 215), or incomplete treatment information (n = 19). Table 1 shows the demographic and clinical parameters of patients with uncomplicated *Plasmodium falciparum* malaria who were included in this analysis.

**Table 1:** Baseline characteristics of patients included in the analysis

Parameter	Median (range) [n or n/N]
Age (years)	10 (0.1–70) [6970]
Parasitaemia (/µL)	70,336 (1000–2,285,920) [6966]
Temperature (°C)	38.0 (34.1–41.5) [3266]
Haemoglobin (g/dL)	12.4 (2.1–19.9) [1812]
Haematocrit (%)	35.0 (11.3–50) [3251]
Anaemia^a^	
None	59 [2359/3966]
Moderate	36 [1422/3966]
Severe	5 [185/3966]
Fever^b^	66 [2164/3266]
Gametocytaemia	32 [1610/5045]
Female	35 [2417/6876]

^a^Defined according to WHO guidelines [23]. For studies where only haematocrit was measured, the following relationship was used to estimate haemoglobin concentration: Haematocrit = 5.62 + 2.60 × Haemoglobin [24]

^b^Defined as axillary temperature >37.5 °C

Patients were treated with (a) artesunate (AS) alone (n = 842); (b) AS alone in the first 3 days or longer followed by a standard ACT: artemether-lumefantrine (AL); artesunate-amodiaquine (ASAQ); artesunate-mefloquine (ASMQ); or dihydroartemisinin-piperaquine (DP) (n = 2751); (c) AL (n = 2217); (d) DP (n = 55); (e) ASMQ, with the first dose of MQ administered at a median (range) of 46 (0–71) hours (n = 1343); or (f) artesunate-chlorproguanil-dapsone (n = 914). There were also 341 hyperparasitaemic patients studied in Thailand (Study ID 1) who received AS together with either doxycycline or clindamycin.

The target daily dose of AS varied between 2 (n = 862, 24 %), 4 (n = 2,544, 71 %), 6 (n = 119, 3 %), and 8 (n = 66, 2 %) mg/kg, with patients in Cambodia receiving the higher doses of 6 or 8 mg/kg in one study. In two studies the initial dose of AS was higher than on subsequent days: in hyperparasitaemic patients in Thailand (Study ID 1: 4 mg/kg followed by 2 mg/kg, n = 2509) and in patients in Mali (Study ID 14: 6 or 4 mg/kg followed by 2 mg/kg, n = 100). Seven studies (Study IDs: 2, 6, 10, 11, 13, 17, 24) at 14 locations randomized 1242 patients to either 2 mg/kg or 4 mg/kg daily doses of AS, alone or in combination with an ACT given at 72 h.

### Estimates of parasite clearance

Among 8536 patients, 6975 (82 %) had sufficient parasite counts taken for PCE estimation of PC_1/2_. The majority of the excluded patients came from three studies with variable sampling schemes (59 %, Study IDs 4, 20, 21) and from the study with hyperparasitaemic patients (36 %, Study ID 1), which for 10 years routinely recorded parasitaemia every 6 h until clearance.

Only two positive parasite counts were used to estimate PC_1/2_ in 878 patients, either because only two positive measurements were available (n = 844) or measurements were excluded as being part of the lag or tail phases (n = 34). For these profiles with only two positive parasite counts available, the PCE replaces the first zero count with the detection limit [7] and the resulting PC_1/2_ estimate clearly overestimates the true PC_1/2_. However, the estimated PC_1/2_ was still considered informative as 25 % (214/844) of these profiles had estimated values <2 h and 73 % (618/844) had estimated values <3 h, indicating that parasite clearance in these patients was rapid and thus provided no evidence for artemisinin resistance. Of the remaining patients, 73 % (165/226) with an estimated PC_1/2_ >3 h had an initial parasitaemia <10,000 parasites/µL and 77 % (175/226) had parasite counts measured using one of the twice-daily schemes. For 21 % (1489/6975) of profiles, a non-zero lag phase was estimated with median (range) duration of 6 (1.5–60) hours, with 6 % (90/1489) having a lag phase duration >12 h.

The median (range) goodness of fit statistic, pseudo-R^2^, was 0.938 (−198 to 0.999), with 89 % (6197/6975) of profiles having a pseudo-R^2^ >0.8. Only 0.9 % (65/6975) of profiles had a negative pseudo-R^2^, indicating that the model was not a good representation of the data.

The 95 % confidence interval (CI) for the estimated PC_1/2_ was wide for 11 % (740/6975) of profiles; the 95 % CI either included negative values or the upper limit was greater than twice the PC_1/2_ estimate. Of these, 70 % (519/740) were for patients with only two positive parasite counts available.

For the distribution of PC_1/2_ by location, treatment and study year see Additional file 3. See Additional file 4: Table S3 for summaries of PC_1/2_ and other parasitological measures by location and treatment and Additional file 5: Table S4 for proportion of profiles with PC_1/2_ longer than 3, 4, 5 and 6 h.

### Areas with slow parasite clearance

Delayed parasite clearance was observed at sites in Cambodia, Thailand, Myanmar, and Vietnam. For all treatments and locations, the longest PC_1/2_ were observed in three western Cambodian sites: Pailin, Tasanh and Pursat where data from 2007 to 2012 were available; study median PC_1/2_ ranged from 5.6 to 6.7 h, and the proportion of PC_1/2_ >5 h ranged from 61 to 80 %. Importantly, no significant trend of increasing PC_1/2_ was observed at these sites over that time interval. At the two other Cambodian sites, Ratanakiri and Preah Vihear, parasite clearance was significantly faster (p < 0.001; median PC_1/2_ of 3.0 and 3.8 h, and proportion of PC_1/2_ >5 h of 4 and 22 %, respectively) and also different between these two sites (p = 0.011).

In contrast, a disproportionate increase in PC_1/2_ was observed in western Thailand after 2003 (p < 0.001, fractional polynomials), with an average increase in PC_1/2_ of 7.1 % (95 % CI 5.7–8.6) per year after 2005. The PC_1/2_ values (p = 0.247) and changes in PC_1/2_ over time (p = 0.628) were similar in hyperparasitaemic and uncomplicated falciparum malaria patients from 2008 to 2011 (Additional file 3: Figure S2). Overall, the proportion of PC_1/2_ <3 h decreased from 67 % (n = 169) in 2003 to 11 % (n = 75) in 2012, and the proportion of PC_1/2_ >5 h increased from 6 to 55 % during this time period (Additional file 4: Table S4).

### Areas with rapid parasite clearance

**Fig. 3:**
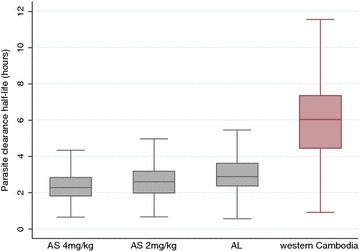
Distribution of PC_1/2_ by treatment group in areas with artemisinin-sensitive parasite population. Figure in *red* shows PC_1/2_ values in all western Cambodian sites for comparison

**Fig. 4:**
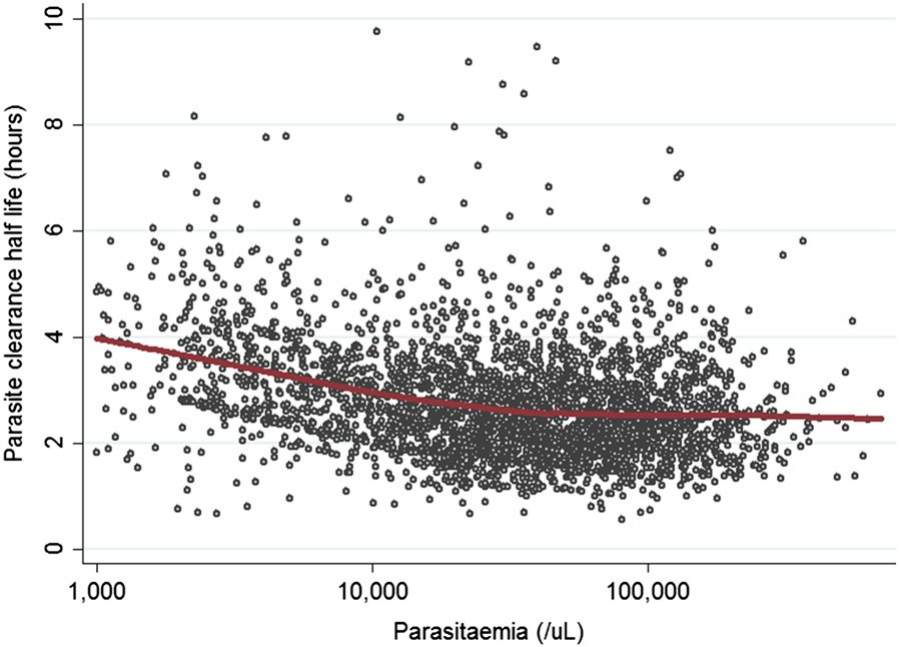
Relationship between initial parasitaemia and estimated PC_1/2_ in patients in areas with artemisinin-sensitive parasite populations. *Red line* shows locally weighted scatterplot smoothing estimator (LOWESS)

Among studies in areas with artemisinin-sensitive parasites (n = 3208), patients who received AS 2 mg/kg (with or without partner drugs) or the standard six-dose AL regimen had longer PC_1/2_ values compared to patients who received AS 4 mg/kg (with or without partner drugs) by 7.3 % (95 % CI 1.9–12.9, p = 0.007) and 7.4 % (95 % CI 3.8–11.2, p < 0.001), respectively (Fig. 3). These comparisons are adjusted for study site and study design characteristics which affect PC_1/2_ estimates: (a) patients with twice-daily sampling (Study IDs 4, 13, 20, 21) had 16.2 % (95 % CI 7.6–25.6) longer PC_1/2_ compared to those with more frequent schedules (p < 0.001); (b) patients with insufficient number of data points to estimate lag phases had 31 % (95 % CI 26–37) longer PC_1/2_ than those with sufficient data (p < 0.001). Since patients with very low initial parasitaemias and short PC_1/2_ are excluded from the analysis because of insufficient data, this creates a negative association between the initial parasitaemia and PC_1/2_ (Fig. 4). Mean PC_1/2_ was estimated to decrease by 16 % (95 %CI 15–18) per tenfold increase in parasitaemia.

In artemisinin-sensitive areas, profiles with a lag phase had 6.4 % (95 % CI 3.6–9.1) shorter PC_1/2_ compared to profiles without a lag phase (p < 0.001, adjusted for all the above factors). No association was observed between the duration of the lag phase and PC_1/2_ among 687 patients with a non-zero lag phase (p = 0.220, adjusted for the above factors).

### Initial parasitaemia

Studies differed in their admission parasitaemia inclusion criteria. It was assumed that the log-transformed initial parasitaemias followed a truncated normal distribution with lower and upper truncation consistent with the inclusion criteria. In all but five studies, there was no evidence against this assumption of truncated normality (p values ranged from 0.17 to 0.98); the exceptions were four multi-centre and/or multi-country studies (Study IDs 4, 21, 22, 23) and a study with two distinct age groups (Study ID 20; young children weighing 12–20 kg and older children weighing 20–40 kg). For these five studies, there was clear evidence of a multimodal distribution of initial parasitaemias.

### Parasite clearance and clinical covariates

#### Areas with rapid parasite clearance

**Fig. 5:**
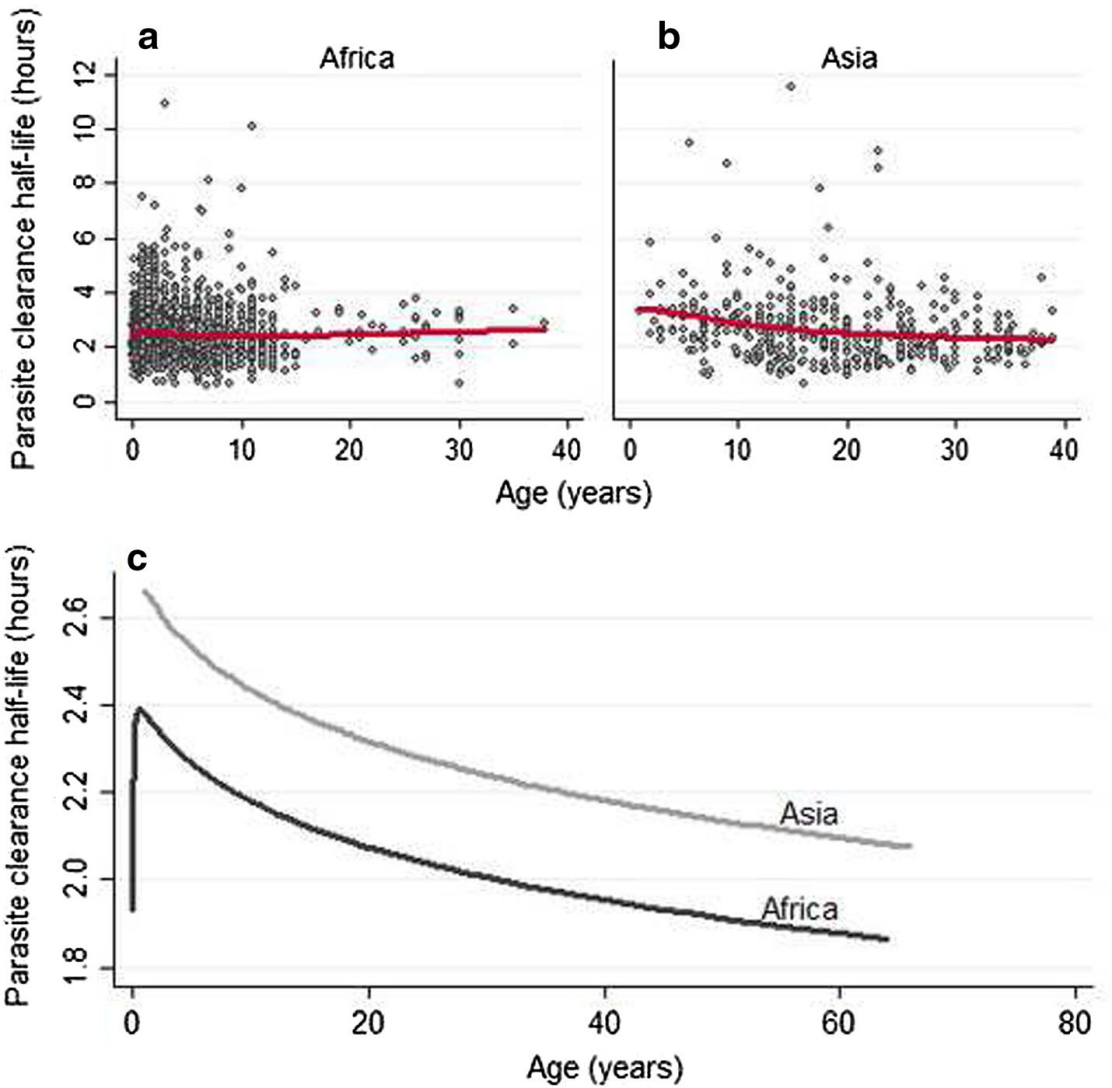
Relationship between patient age and PC_1/2_ in patients in areas with artemisinin-sensitive parasites. (1) Observed data in Africa (**a**) and Asia (**b**) with *red line* showing locally weighted scatter-plot smoothing estimator (LOWESS); only patients with 6-h sampling and enough data points for the full Parasite Clearance Estimator model to be fitted are presented; (2) predicted relationship from multivariate model using fractional polynomials (**c**); adjusted for treatment group, region, initial parasitaemia, presence of lag phase and study design characteristics

In artemisinin-sensitive areas (all countries in Africa, Laos, Bangladesh, Thailand before year 2000, Ratanakiri in Cambodia; n = 3208), after adjusting for study design factors, patient age and treatment were associated independently with PC_1/2_. Adjusting for age changed the treatment effect very little. Patients who received 2 mg/kg AS or AL had 7.2 % (95 % CI 1.8–12.8) and 7.3 % (3.7–11.0) longer PC_1/2_, respectively, compared to patients who received 4 mg/kg AS (p ≤ 0.008). Young children cleared parasites more slowly than older patients: PC_1/2_ was 11.3 % (95 % CI 2.6–20.8, p = 0.010) longer in infants aged <1 year and 9.4 % (95 % CI 3.5–15.7, p = 0.002) longer in children aged 1–4 years compared to older patients (Fig. 5a, b). There was no significant difference in PC_1/2_ between children aged 5–14 years and adults (p = 0.129). The relationship between patient age and PC_1/2_ was examined further in the multivariate model (Fig. 5c). After adjusting for age and treatment, higher parasitaemia remained associated with lower estimates of PC_1/2_ (by a 17 % (95 % CI 15–18) per tenfold increase in parasitaemia). Other factors, examined on a subset of patients with available data, were independently associated with longer PC_1/2_: fever (7.0 %, 95 % CI 3.2–10.8, p < 0.001, n = 1636); severe anaemia (13.5 %, 95 % CI 6.4–21.1, p < 0.001, n = 2043) and moderate anaemia defined as haemoglobin level from 7 to 9 g/dL (4.3 %, 95 % CI 1.0–7.7, p = 0.010, n = 2043). No associations between PC_1/2_ and gametocyte carriage, transmission intensity or nutritional status of children were observed. A lag phase was detected more frequently in patients receiving AL (OR = 2.14, 95 % CI 1.29–3.59 compared to other treatments, p = 0.004), high initial parasitaemia (OR = 1.77, 95 % CI 1.28–2.45 per tenfold increase, p = 0.001) or fever (OR = 1.63, 95 % CI 1.21–2.21 compared to patients presenting without fever, p = 0.001). Among 1297 patients treated with AL [median (range) daily artemether dose 2 (0.9–4) mg/kg], no significant association was found between PC_1/2_ and artemether dose. In contrast, none of the patient covariates or treatments were associated with a risk of PC_1/2_ being >5 h.

#### Areas with slow parasite clearance

In areas with previously documented slow parasite clearance rates, no significant association between PC_1/2_ and patient age was observed. After adjusting for study design factors, admission gametocytaemia was associated with an 11.1 % (95 % CI 5.5–16.9, p < 0.001, n = 3574) increase in PC_1/2_, and temperature >37.5 °C was associated with a 7.3 % (95 % CI 1.3–13.8, p = 0.017, n = 1491) increase in PC_1/2_. The relationship between PC_1/2_ and initial parasitaemia was the opposite of that in the artemisinin-sensitive population: a tenfold increase in parasitaemia was associated with a 5.2 % (95 % CI 0.7–9.9, p = 0.024, n = 3574) increase in PC_1/2_ when adjusted for study site.

#### Artemisinin dose and PC_1/2_

**Fig. 6:**
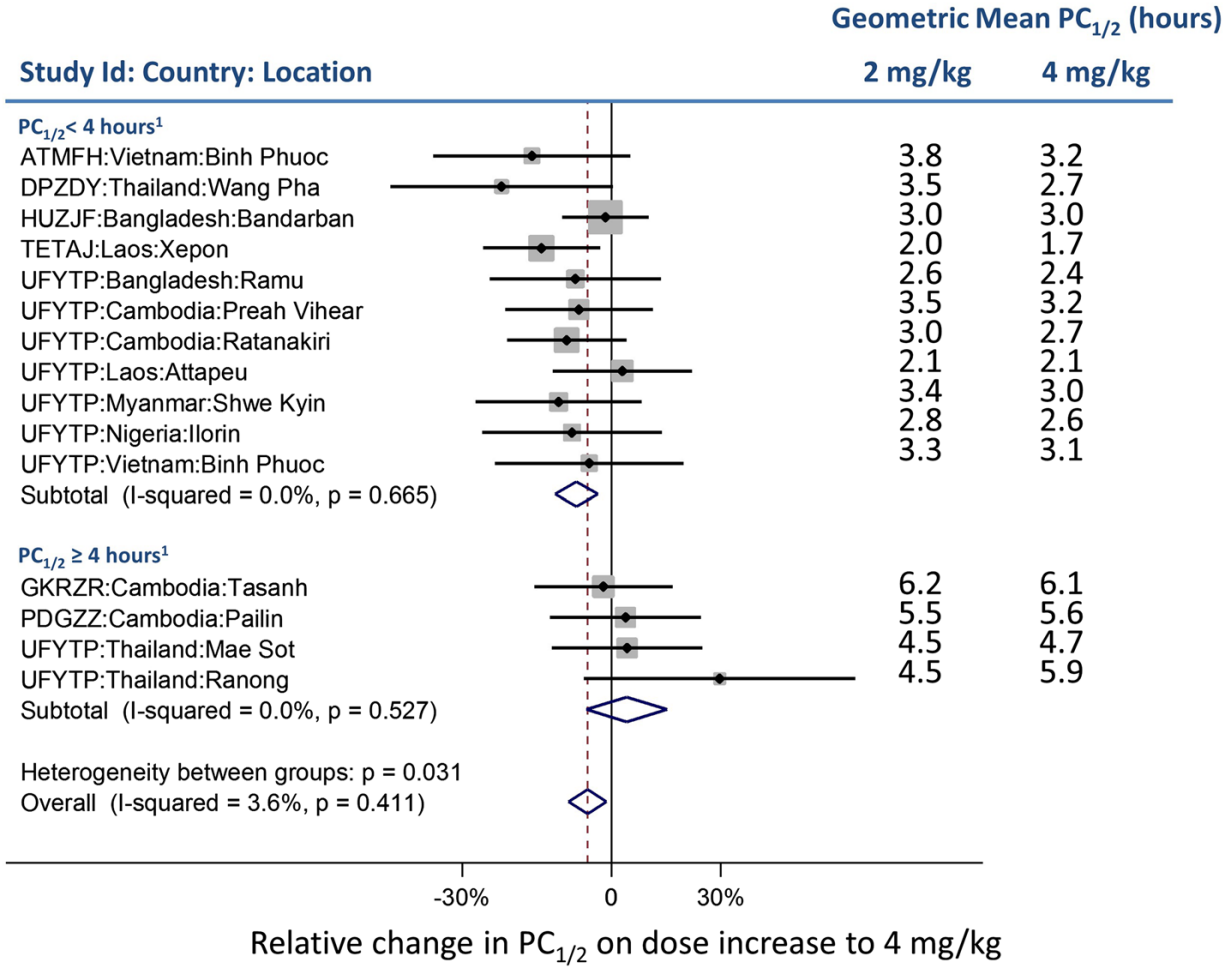
Meta-analysis of dose effect in randomized studies with artesunate alone in the first 72 h. ^1^geometric mean of PC_1/2_ in 2 mg/kg treatment arm

Six studies at 15 locations had randomized AS treatment arms of 2 and 4 mg/kg. The higher dose was associated with an 8.1 % (95 % CI 3.2–12.6, p = 0.001) decrease in PC_1/2_ in sites with geometric mean PC_1/2_ <4 h (in AS 2 mg/kg dose arm), whereas there was no significant (p = 0.455) difference in PC_1/2_ in the remaining sites with geometric mean PC_1/2_ ≥4 h. Overall change was estimated as −5.5 % (95 % CI −9.7 to −1.2, p = 0.013) (test for heterogeneity between groups, p = 0.031) (Fig. 6).

#### Treatment outcome

Among 3328 patients with defined outcome, 93 (2.8 %) had PCR-confirmed recrudescences by day 63. After adjusting for study design factors in a multivariate model, longer PC_1/2_ was associated with an increased risk of recrudescence: HR = 2.91, 95 % CI 1.92–4.31, for a doubling of PC_1/2_, p < 0.001). Patients with high initial parasitaemia also had a higher risk of recrudescence: HR = 2.23, 95 % CI 1.44–3.46, for a tenfold increase in parasitaemia, p < 0.001. No significant interaction between PC_1/2_ and initial parasitaemia was detected. After adjusting for the initial parasitaemia, PC_1/2_ and parasite sensitivity status, the recrudescence rates varied between regimens with different partner drugs or time of their administration. Recrudescence rates were significantly higher in patients receiving artesunate-chlorproguanil-dapsone than any other ACT (HR = 3.62, 95 %CI 1.74–7.52, p = 0.001) Recrudescence rates were significantly lower in patients receiving AS for 3 days followed by a standard ACT at 72 h (HR = 0.28, 95 % CI 0.11–0.74, p = 0.010) than in all other patients. Other baseline covariates, as well as the presence or duration of lag and tail phases in the parasite clearance curve, were not associated with treatment outcome.

## Discussion

The rate at which asexual *P. falciparum* parasites are cleared from the blood following treatment is the best measure of the anti-malarial effect of artemisinin and its derivatives. This is assessed from the linear component of the log-linear decline in parasite densities and is expressed conveniently as PC_1/2_ [1]. Resistance to artemisinins results in prolongation of the PC_1/2_. This pooled analysis combines the largest set of data, collected in 24 studies over 18 years, from nearly 7000 patients with uncomplicated falciparum malaria in whom frequent measurements of parasitaemia were made. The reference PC_1/2_ estimates provided for 46 locations across Africa and Asia are essential comparators for the early recognition of emerging resistance, and so will be updated continuously as others join the WWARN [44] collaborative effort and provide relevant data sets. An important output of this analysis is that there was no evidence for worsening of artemisinin resistance in western Cambodia. There is substantial concern that failure to eliminate falciparum malaria in this area, the ‘cradle of antimalarial drug resistance’, will lead to higher levels of artemisinin resistance, rendering ACTs progressively less effective. While further worsening of the degree of artemisinin resistance fortunately has not happened, at least until 2012, continued monitoring is vital.

This large dataset allowed estimation of the additional contributions of patient characteristics and study design to parasite clearance estimates, information that is crucial in interpreting and monitoring changes in these estimates, and attributing them to true artemisinin resistance rather than the effects of partner drugs, study design or patient characteristics. The recent discovery [4] and validation [15] of the molecular marker *kelch13* in the Greater Mekong area and the development of suitable in vitro sensitivity tests [6, 45] provide important information. Data from in vitro ring-stage survival assays do reflect artemisinin resistance in vivo, but their use is likely to be limited to few resourced laboratories and thus unlikely to provide comprehensive surveillance information across endemic countries. Mutations in *kelch13* above position 440 correlate with slow parasite clearance rates in the Greater Mekong area, but have not yet been associated with slow rates elsewhere, and cannot yet substitute for PC_1/2_ values as definite measures of clinical artemisinin resistance.

Estimation of PC_1/2_ requires sufficient quality-assured serial parasite blood counts for analysis. In this very large series, the most common problem encountered (13 %) was that only two positive counts were available because of rapid parasite clearance and low initial parasitaemias. Other problems encountered (10 %) were a very long lag phase, large variations in parasite counts resulting in poor fits or large confidence intervals around the estimate. These were most likely a consequence of inaccurate microscopy counts. The initial parasitaemia and frequency of sampling had the greatest effects on the PC_1/2_ estimates, which accords with results of a previously reported simulation study [18]. Ideally, only patients with initial parasitaemia >10,000 parasites/µL should be included in PC_1/2_ assessments. In patients with only two positive parasite counts, estimated PC_1/2_ should be interpreted with caution as it is likely to be overestimated. This is because the lag phase cannot be evaluated and the first recorded zero parasitaemia is treated as a parasite density at the detection limit (so the worst case scenario is assumed). Profiles for which the lag or tail phases were identified, and after their exclusion only two data points were left for the PC_1/2_ estimation, should be excluded from analysis as they likely represent limitations in microscopy-based parasite counting.

A lag phase was detected more frequently in patients presenting with fever, possibly because of the association of fever with synchronous schizont rupture. The more frequent lag phase with AL treatment may result from the initial lower dose and slower absorption and conversion to DHA of oral artemether compared to oral artesunate [46]. Patients with profiles beginning with a lag phase may have had more rapid clearance in the log-linear part of the parasitaemia-time curve (lower PC_1/2_); however, the difference was rather small (6.4 %, 95 % CI 3.6–9.1). This is an artefact of the way the model is fitted—as the lag phase is defined only if the initial clearance is slower and the ratio of the clearance rates between this initial period and the rest of the parasitaemia profile reaches a pre-specified cut-off. Some of the observed differences in slopes are caused by random variation of the microscopy measurement. Excluding this randomly occurring slower (but not faster) part of the profile will result in the overestimation of PC_1/2_ in profiles with a detected lag phase. This phenomenon was observed in 3–10 % of simulated parasite profiles (using previously described methodology [18]), from distributions of PC_1/2_ with mean of two to 6 h and standard deviation (log scale) from 0.05 to 0.3.

The treatment, clinical and demographic variables studied had modest effects on PC_1/2_ estimates, all resulting in less than 20 % change in PC_1/2_, and none associating with an increased risk of PC_1/2_ being >5 h in the rapid-clearing parasite populations.

In areas with artemisinin-sensitive parasite populations, parasite clearance was faster in patients receiving the 4 mg/kg dose of AS than in those receiving the 2 mg/kg dose, which was a robust finding confirmed in meta-analysis performed in a subset of randomized studies as well as in a multivariate analysis of studies with either of the doses administered. It is therefore expected that there will be marked differences between the various currently available ACTs, including AL, ASAQ, DHA-PQP and ASMQ, depending on the dose of artemisinin derivative. However, after adjusting for the sampling scheme, the proportions of patients with PC_1/2_ estimates >5 h were not significantly different between treatments in this study and ranged from 0 to 10 % for studies with six-hourly sampling, and from 0 to 7 % after exclusion of profiles with pseudo R^2^ statistic <0.8.

Therapeutic responses in malaria are enhanced by immunity [1]. As expected from previous work [47, 48], young children had slower parasite clearance rates compared to older patients. However, this was observed only in artemisinin-sensitive parasite populations, with most data coming from Africa. Resistant parasite populations, present only in Southeast Asia, did not demonstrate an age effect. The lack of an age effect on PC_1/2_ could be due to one or more of the following factors: lower background immunity in those patients from low transmission settings, different age distributions studied with 70 % of patients being older than 12 years, nonlinear negative age effect on PC_1/2_ (Fig. 5), or a qualitative pharmacodynamic difference in that whereas most of the clearance of artemisinin-sensitive parasites results from clearance of ring-stage parasites in low transmission settings, in artemisinin-resistant infections cytoadherence becomes a more important contributor to the initial decline in parasitaemia (as it is following quinine treatment) [1]. In both populations, the presence of fever on admission was associated with longer PC_1/2_. This has also been reported in other studies from Kenya [49] and Uganda [47] and may be a surrogate marker of a less effective host immune response. Also, fever in malaria is thought to be caused partly by TNF and other pyrogenic cytokines released as part of the human immune response to products of schizont rupture [50, 51].

The relationship between PC_1/2_ and parasitaemia was different between the sensitive and resistant parasite populations. In sensitive areas, high parasitaemia was associated with shorter PC_1/2_ largely because patients with low initial parasitaemias and rapid clearance are not included in the analysis. In resistant populations with longer PC_1/2_, high parasitaemias were associated with slightly longer PC_1/2_ (by 5.2 % per tenfold increase).

The main limitation of this analysis is the heterogeneity in study designs and treatments which did not permit a more detailed examination of treatment and dose effects, as they were confounded by the exclusion of patients with relatively low initial parasitaemias, different partner drugs, and different timings and frequencies of sampling.

## Conclusion

This pooled analysis showed that the main factor affecting estimates of parasite clearance is the study design—relatively low initial parasitaemia resulting in too few data points to estimate the clearance accurately, and too infrequent sampling. Additionally, in artemisinin-sensitive parasite populations, PC_1/2_ is affected by artemisinin dose, patient age and the presence of fever as likely surrogates of acquired immunity. Therefore, it is important to consider these factors in early surveillance of changes in parasite sensitivity. This pooled analysis provides critical baseline information to monitor future evolution of PC_1/2_ in malaria endemic countries.

## Additional files

**Additional file 1: Table S1.** Methods used for counting parasites. Summary of the methodologies used in individual studies to measure parasitaemia.
**Additional file 2: Table S2.** Description of 24 studies included in the analysis. Description of studies included in the analysis with respect to location and year, treatment administered and study population.
**Additional file 3: Figures S1-S5.** Distribution of PC_1/2_ by study location, treatment and year. Box plots of PC_1/2_ by study location, treatment and study year.
**Additional file 4: Table S3.** Summary of parasitological measures I: PC_1/2_, PC50 and PC90 summarized by study location, year and treatment.
**Additional file 5: Table S4.** Summary of parasitological measures II: Proportion of profiles with PC_1/2_ above cut-off of 3, 4, 5 and 6 h presented by study location, year and treatment.
