# Supplementary material for: Baseline data of parasite clearance in patients with falciparum malaria treated with an artemisinin derivative: an individual patient data meta-analysis
Source: Malar J. 2015 Sep 22;14:359. doi: 10.1186/s12936-015-0874-1 (PMC4578675; doi:10.1186/s12936-015-0874-1)
Supplement: Supplementary file 1 — Additional file 1: Table S1. Methods used for counting parasites. Summary of the methodologies used in individual studies to measure parasitaemia. [file 12936_2015_874_MOESM1_ESM.docx]

**Table S1 Methods used for counting parasites**

| Study # | Study ID | Country | Location | Parasitaemia counting method  Inclusion criteria Counting method^1^ Sampling schedule^2^ | | |
| --- | --- | --- | --- | --- | --- | --- |
| 1 | MRGRH | Thailand | Western border | >185,000 | 1000rbc, 500wbc | SS1 |
| 2 | DPZDY | Thailand | Wang Pha | >10,000 | 1000rbc, 200wbc | SS3 |
| 3 | QBPQM | Thailand | Wang Pha | 10,000-175,000 | 1000rbc, 200wbc | SS2 |
| 4 | EFTTU | Thailand | Multiple sites | >500 | 1000rbc, 500wbc | SS6^3^ |
| 5 | GHNKU | Thailand | Bangkok | >500 | 1000rbc, 500wbc | SS5 |
| 6 | PDGZZ | Cambodia | Pailin | >10,000 | 1000rbc, 200wbc | SS2 |
| 7 | FARTM | Cambodia | Pailin | 10,000-175,000 | 1000rbc, 200wbc | SS2 |
| 8 | PDKJM | Cambodia | Pursat | >10,000 | 200wbc | SS1 |
| 9 | PDKJM | Cambodia | Ratanakiri | >10,000 | 200wbc | SS1 |
| 10 | GKRZR | Cambodia | Tasanh | 1,000-200,000 | 5000rbc, 200wbc | SS2 |
| 11 | TETAJ | Laos | Savannakhet | 10,000-175,000 | 1000rbc, 500wbc | SS1 |
| 12 | MSDDE | Tanzania | Fukayosi | 2,000-20,000 | 500wbc | SS4 |
| 13 | HUZJF | Bangladesh | Bandarban | 1,000-100,000 | 2000rbc, 200wbc | SS6 |
| 14 | BYMYG | Mali | Kenieroba | 10,000-100,000 | 300wbc | SS1 |
| 15 | SRDFP | Mali | Sikasso | 2,000-200,000 | 200wbc | SS5 |
| 16 | TZDRS | Kenya | Pingilikani | Unknown | Unknown | SS1 |
| 17 | ATMFH | Vietnam | Binh Phuoc | 10,000-100,000 | 1000rbc, 400wbc | SS1 |
| 18 | NKTYE | Vietnam | Phuoc Chien | 200 - 200,000 | 200wbc | SS6 |
| 19 | PNUNE | Uganda | Mbarara | <250,000 | 1000rbc, 500wbc | SS1 |
| 20 | ADXZX | Gabon | Lambaréné | 1,000-250,000 | Unknown | SS7 |
| 21 | CXJYT | Multiple countries | Kenya, Nigeria, Tanzania | 1,000-100,000 | Unknown | SS8 |
| 22 | EDPJN | Multiple countries | Benin, Kenya, Mali, Mozambique, Tanzania | 2,000-20,000 | 200wbc | SS8^4^ |
| 23 | MEFSC | Multiple countries | Burkina Faso, Ghana, Kenya, Nigeria, Tanzania | 2,000-200,000 | Unknown | SS5 |
| 24 | UFYTP | Multiple countries | Cambodia, Thailand, Laos, Vietnam, Myanmar, Bangladesh, India, Nigeria, Kenya, Democratic Republic of Congo | 10,000-200,000 | 1000rbc, 500wbc | SS1 (+ 4 and 8) |

^1^ Xrbc = parasites counted per X red blood cells (thin smear), Xwbc = parasites counted per X white blood cells (thick smear); ^2^Sampling schedules: 6 hourly until negative (SS1); 0,2,4,6,8,12, then 6 hourly until negative (SS2); 0,4,8,12, then 6 hourly until negative (SS3); 0,2,4,8,16,24, then 12 hourly until negative (SS4); 8 hourly until negative (SS5); 12 hourly until negative (SS6); 0,2,4,6,12, then 12 hourly until negative (SS7); 0,8,24, then 12 hourly until negative (SS8). ^3^Irregular; ^4^Up to 60 hours, irregular.
