# Supplementary material for: Baseline data of parasite clearance in patients with falciparum malaria treated with an artemisinin derivative: an individual patient data meta-analysis
Source: Malar J. 2015 Sep 22;14:359. doi: 10.1186/s12936-015-0874-1 (PMC4578675; doi:10.1186/s12936-015-0874-1)
Supplement: Supplementary file 2 — Additional file 2: Table S2. Description of 24 studies included in the analysis. Description of studies included in the analysis with respect to location and year, treatment administered and study population. [file 12936_2015_874_MOESM2_ESM.docx]

**Table S2 Summary of the 24 studies in the analysis**

| Study # | Study ID | Country | Location | Study years | Treatment^1^ | Age (years)^2^ | Parasitaemia (/µL)^2^ | N^3^ | Reference |
| --- | --- | --- | --- | --- | --- | --- | --- | --- | --- |
| 1 | MRGRH | Thailand | Western border | 2001-2012 | AS, AS+MQ, AS+antibiotics^3^ | 12 (0-70) | 264,890 (16-2,285,920) | 3,393 | [14] |
| 2 | DPZDY | Thailand | Wang Pha | 2008 | AS+MQ, AS | 29 (19-50) | 30,477 (4,160-304,655) | 40 | [3] |
| 3 | QBPQM | Thailand | Wang Pha | 2009-2010 | AS+MQ | 26 (18-58) | 36,989 (2,032-310,232) | 80 | [10] |
| 4 | EFTTU | Thailand | Multiple sites | 1996 -1997 | AL | 22 (3-62) | 6,862 (290-464,880) | 239 | [42] |
| 5 | GHNKU | Thailand | Bangkok | 1998 | AL | 25 (12-71) | 3,540 (13-436,050) | 219 | [38] |
| 6 | PDGZZ | Cambodia | Pailin | 2007-2008 | AS+MQ, AS | 21 (9-56) | 54,008 (10,550-262,253) | 40 | [3] |
| 7 | FARTM | Cambodia | Pailin | 2009-2010 | AS+MQ | 21 (7-53) | 50,240 (10,299-386,848) | 79 | [10] |
| 8 | PDKJM | Cambodia | Pursat | 2009-2010 | AS+MQ | 24 (10-67) | 63,577 (10,240-546,461) | 198 | [9] |
| 9 | PDKJM | Cambodia | Ratanakiri | 2010 | AS+MQ | 14 (1-60) | 69,333 (10,280-526,000) | 53 | Unpublished |
| 10 | GKRZR | Cambodia | Tasanh | 2008-2009 | AS | 25 (18-62) | 14,097 (1,104-182,785) | 143 | [26] |
| 11 | TETAJ | Laos | Savannakhet | 2010 | AS+AL | 28 (11-66) | 41,134 (10,299-165,792) | 44 | [13] |
| 12 | MSDDE | Tanzania | Fukayosi | 2006 | AL | 4 (1-10) | 43,440 (2,120-200,400) | 50 | [37] |
| 13 | HUZJF | Bangladesh | Bandarban | 2008-2009 | AS | 22 (8-64) | 8,109 (493-94,240) | 101 | [43] |
| 14 | BYMYG | Mali | Kenieroba | 2010-2011 | AS+AQ | 5 (1-15) | 29,050 (2,400-175,325) | 261 | [12] |
| 15 | SRDFP | Mali | Sikasso | 2010-2011 | AS | 6 (1-11) | 26,140 (1,072-289,680) | 100 | [39] |
| 16 | TZDRS | Kenya | Pingilikani | 2010-2011 | AS | 4 (0-10) | 95,203 (16,579-566,400) | 173 | Unpublished |
| 17 | ATMFH | Vietnam | Binh Phuoc | 2010-2011 | AS+DHA+PIP, DHA+PIP | 26 (10-70) | 28,558 (10,048-97,842) | 166 | [11] |
| 18 | NKTYE | Vietnam | Phuoc Chien | 2008-2009 | AS+AQ, ART+PIP | 14 (5-65) | 21,576 (30-162,527) | 128 | [41] |
| 19 | PNUNE | Uganda | Mbarara | 2005 | AL | 4 (0.1-54) | 65,218 (2,663 – 553,507) | 44 | [36] |
| 20 | ADXZX | Gabon | Lambaréné | 2005-2006 | AS+MQ | 6 (1-13) | 37,100 (1,000-249,700) | 71 | [34] |
| 21 | CXJYT | Multiple countries | Kenya, Nigeria, Tanzania | 2002-2003 | AL | 2 (0.2-10) | 18,488 (100-137,760) | 310 | [35] |
| 22 | EDPJN | Multiple countries | Benin, Kenya, Mali, Mozambique, Tanzania | 2006-2007 | AL/ AL dispersible | 3.5 (0.2-12.4) | 29,240 (520-628,571) | 898 | [33] |
| 23 | MEFSC | Multiple countries | Burkina Faso, Ghana, Kenya, Nigeria, Tanzania | 2006-2007 | AL/CDA | 3 (1-14) | 27,828 (50-705,600) | 1,372 | [40] |
| 24 | UFYTP | Multiple countries | Cambodia, Thailand, Laos, Vietnam, Myanmar, Bangladesh, India, Nigeria, Kenya, Democratic Republic of Congo | 2011-1013 | AS + AL/AS+SP/AS+MQ/DHA+PIP | 21 (0.7 – 65) | 52,250 (2,560-605,329) | 1,241 | [15] |

^1^ Treatment: AL = artemether–lumefantrine; AS = artesunate; MQ = mefloquine; CDA = chlorproguanil-dapsone-artesunate; DHA = dihydroartemisinin; PIP = piperaquine;

^2^ Median (range) are given

^3^ N = total number of patients in the study

^4^ clindamycin or doxycycline.
