# Supplementary material for: Baseline data of parasite clearance in patients with falciparum malaria treated with an artemisinin derivative: an individual patient data meta-analysis
Source: Malar J. 2015 Sep 22;14:359. doi: 10.1186/s12936-015-0874-1 (PMC4578675; doi:10.1186/s12936-015-0874-1)

Additional file 3

Distribution of $PC_{1/2}$ by study location and year

Figure S1 Distribution of $PC_{1/2}$ in Cambodia in patients treated with artesunate alone in the first 72 hours or artesunate and mefloquine

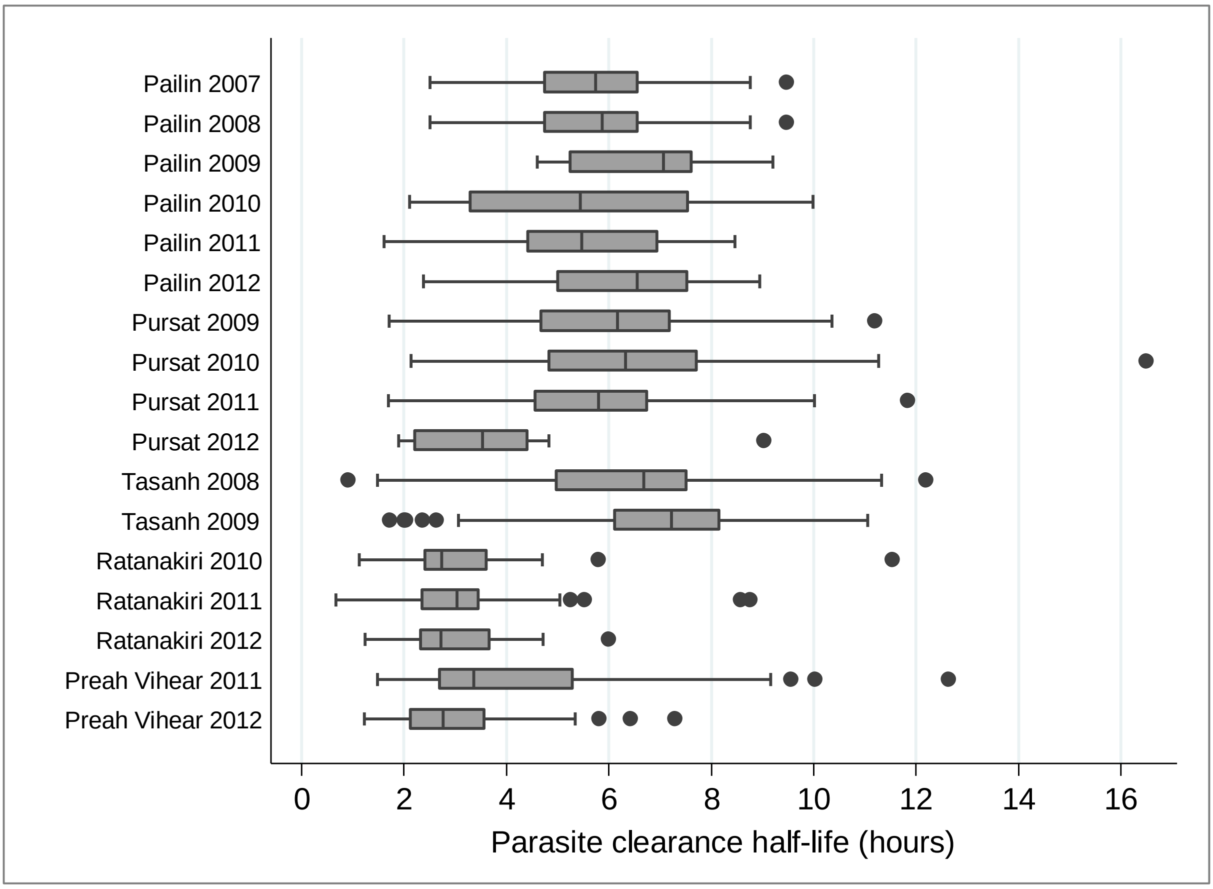

Figure S2. Distribution of $PC_{1/2}$ in western Thailand in patients treated with artesunate alone in the first 48 hours or in combination with mefloquine

Light grey bars represent data from the study in hyperparasitaemic patients, dark grey bars represent data from studies in uncomplicated malaria.

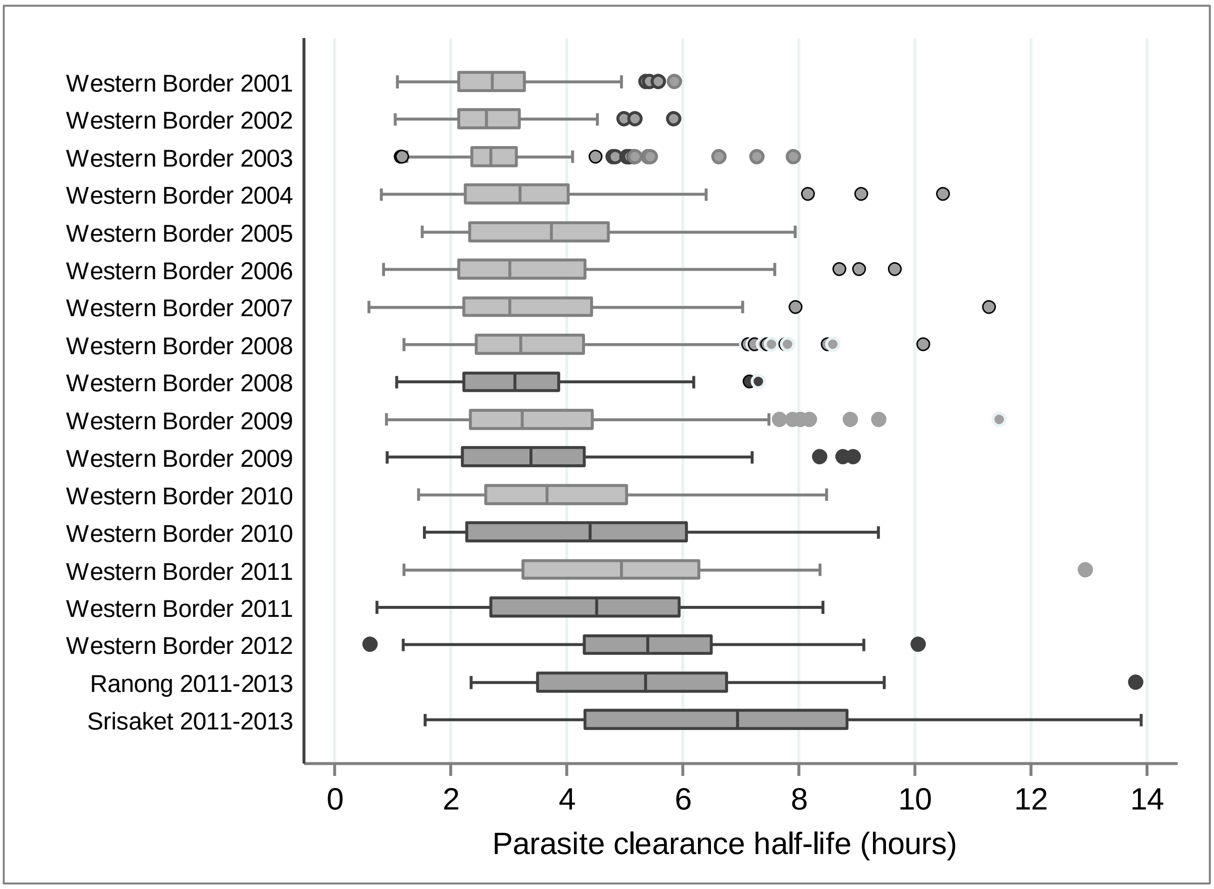

Figure S3. Distribution of $PC_{1/2}$ in patients treated with a standard artemether-lumefantrine regimen

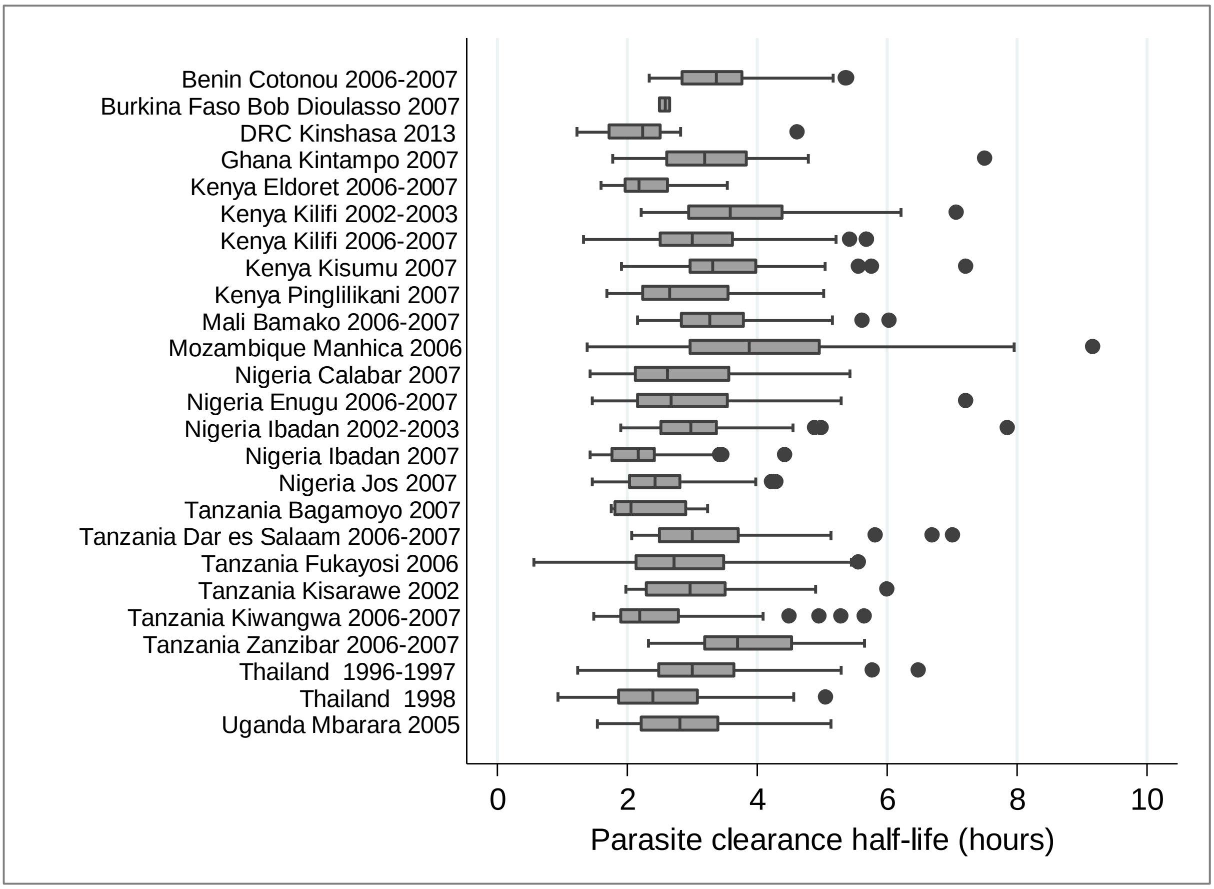

Figure S4. Distribution of $PC_{1/2}$ in patients treated with 2 mg/kg/day artesunate alone or in combination with other drugs (in all areas except Thailand and Cambodia)

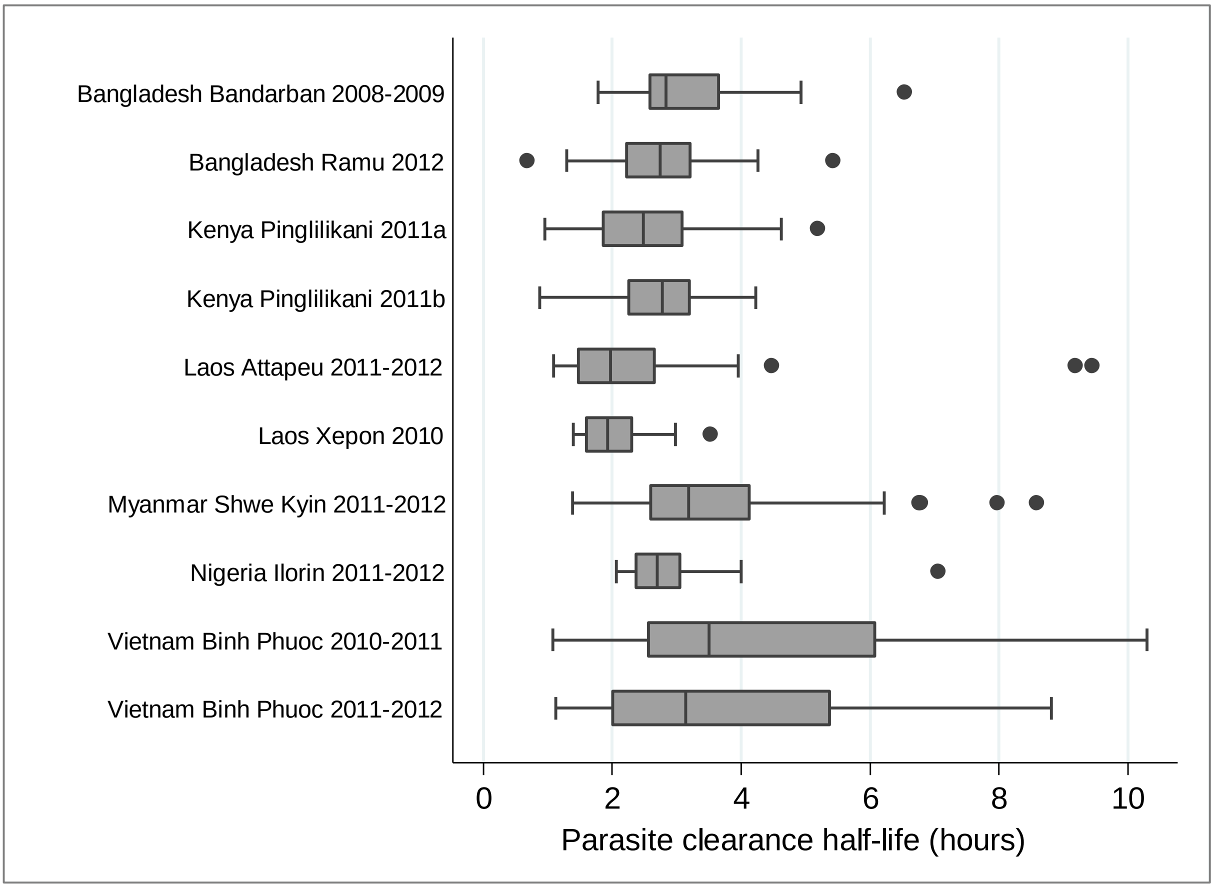

Figure S5. Distribution of $PC_{1/2}$ in patients treated with 4 mg/kg/day artesunate alone or in combination with other drugs (in all areas except Thailand and Cambodia)

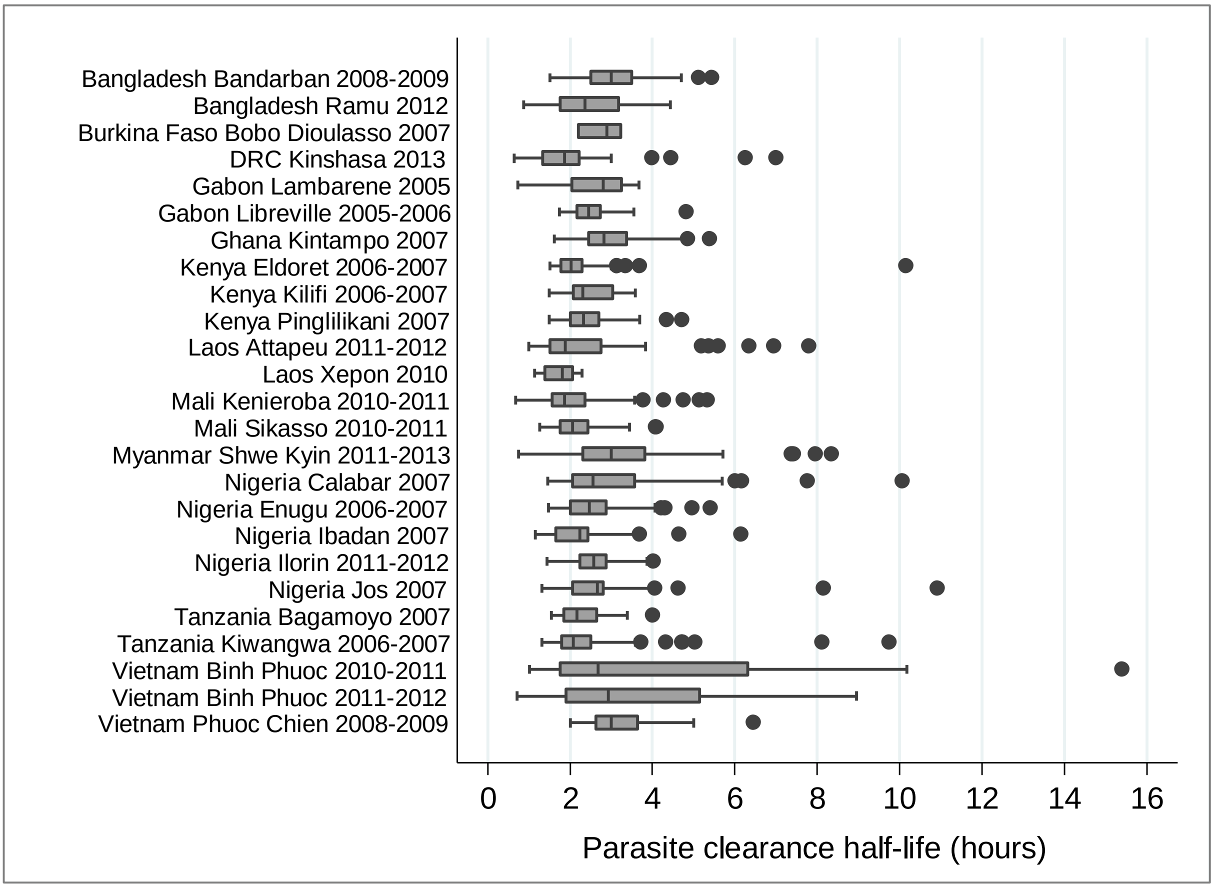

Supplement: Supplementary file 3 — Additional file 3: Figures S1-S5. Distribution of PC1/2 by study location, treatment and year. Box plots of PC1/2 by study location, treatment and study year. [file 12936_2015_874_MOESM3_ESM.pdf]
