# Supplementary material for: Baseline data of parasite clearance in patients with falciparum malaria treated with an artemisinin derivative: an individual patient data meta-analysis
Source: Malar J. 2015 Sep 22;14:359. doi: 10.1186/s12936-015-0874-1 (PMC4578675; doi:10.1186/s12936-015-0874-1)
Supplement: Supplementary file 4 — Additional file 4: Table S3. Summary of parasitological measures I: PC1/2, PC50 and PC90 summarized by study location, year and treatment. [file 12936_2015_874_MOESM4_ESM.docx]

**Table S3 Summary of parasitological measures I: PC_1/2_, PC50 and PC90**

|  | Study ID | Study site | Study years | PC_1/2_ (hours) | | | PC50 (hours) | | PC90 (hours) | |
| --- | --- | --- | --- | --- | --- | --- | --- | --- | --- | --- |
|  |  |  |  | Median (range) | Geo mean (cv)^1^ | N | Median (range) | N | Median (range) | N |
| **AS 2mg/kg**^2^ Bangladesh | HUZJF | Bandarban | 2008-2009 | 2.8 (1.8-6.5) | 3.0 (0.3) | 49 | 5.2 (2.2-20.9) | 51 | 15.1 (4.5-28.5) | 51 |
| Bangladesh | UFYTP | Ramu | 2012 | 2.7 (0.7-5.4) | 2.6 (0.4) | 28 | 5.2 (2.2-10.4) | 28 | 11.1 (4.0-22.4) | 28 |
| Cambodia | PDGZZ | Pailin | 2007 | 5.6 (2.5-8.6) | 5.5 (0.3) | 20 | 7.0 (1.5-17.2) | 18 | 21.3 (7.9-45.3) | 20 |
| Cambodia | GKRZR | Tasanh | 2008-2009 | 7.0 (0.9-11.3) | 6.2 (0.5) | 75 | 8.4 (1.2-31.6) | 75 | 20.3 (2.8-55.5) | 75 |
| Cambodia | UFYTP | Preah Vihear | 2011-2012 | 3.3 (1.4-10.0) | 3.5 (0.5) | 60 | 7.6 (2.3-18.6) | 59 | 16.2 (4.3-56.8) | 60 |
| Cambodia | UFYTP | Ratanakiri | 2011-2012 | 3.1 (0.7-8.8) | 3.0 (0.5) | 60 | 6.4 (2.0-14.6) | 57 | 15.6 (3.6-36.3) | 58 |
| Kenya | TZDRS | Pingilikani | 2011 | 2.5 (1.0-5.2) | 2.4 (0.4) | 155 | 7.2 (3.0-27.4) | 165 | 11.8 (5.4-33.8) | 166 |
| Kenya | UFYTP | Pingilikani | 2011 | 2.8 (0.9-4.2) | 2.5 (0.3) | 57 | 5.3 (1.3-13.0) | 50 | 11.6 (3.4-27.5) | 54 |
| Laos | TETAJ | Savannakhet | 2010 | 1.9 (1.4-3.5) | 2.0 (0.2) | 22 | 7.6 (1.5-19.2) | 22 | 13.2 (6.7-28.3) | 22 |
| Laos | UFYTP | Attapeu | 2011-2012 | 2.0 (1.1-9.5) | 2.1 (0.5) | 58 | 8.1 (2.5-16.3) | 57 | 14.8 (5.5-28.5) | 56 |
| Myanmar | UFYTP | Shwe Kyin | 2011-2012 | 3.2 (1.4-8.6) | 3.4 (0.4) | 40 | 7.7 (2.4-19.8) | 40 | 17.3 (5.3-39.7) | 39 |
| Nigeria | UFYTP | Ilorin | 2011-2012 | 2.7 (2.1-7.1) | 2.8 (0.3) | 17 | 7.9 (2.7-22.0) | 13 | 17.5 (6.8-33.3) | 16 |
| Thailand | UFYTP | Mae Sot | 2011-2012 | 5.0 (0.6-10.1) | 4.5 (0.6) | 58 | 8.1 (2.0-27.5) | 54 | 21.8 (3.7-50.4) | 55 |
| Thailand | UFYTP | Ranong | 2011-2012 | 5.3 (2.4-6.3) | 4.5 (0.4) | 10 | 8.0 (2.9-18.9) | 9 | 29.7 (13.0-38.7) | 9 |
| Thailand | DPZDY | Western border | 2008 | 3.4 (2.0-7.3) | 3.5 (0.4) | 20 | 5.3 (2.1-24.6) | 20 | 12.0 (3.8-42.2) | 20 |
| Vietnam | ATMFH | Binh Phuoc | 2010-2011 | 3.5 (1.1-10.3) | 3.8 (0.6) | 55 | 10.5 (3.2-52.5) | 55 | 18.1 (5.8-84.2) | 55 |
| Vietnam | UFYTP | Binh Phuoc | 2011-2012 | 3.1 (1.1-8.8) | 3.3 (0.6) | 59 | 8.9 (2.2-33.3) | 57 | 16.5 (3.9-53.3) | 59 |
| **AS 4mg/kg**^2^  Bangladesh | HUZJF | Bandarban | 2008-2009 | 3.0 (1.5-5.4) | 3.0 (0.3) | 48 | 5.9 (1.9-20.0) | 50 | 15.1 (3.4-25.5) | 50 |
| Bangladesh | UFYTP | Ramu | 2012 | 2.4 (0.9-4.4) | 2.4 (0.4) | 27 | 3.9 (2.1-12.1) | 27 | 7.7 (3.7-18.8) | 27 |
| Burkina Faso | MEFSC | Bobo-Dioulasso | 2007 | 2.9 (2.2-3.2) | 2.7 (0.2) | 3 | 11.2 (10.5-12.5) | 3 | 15.3 (14.8-15.8) | 3 |
| Cambodia | PDGZZ | Pailin | 2007 | 5.9 (2.6-9.5) | 5.6 (0.3) | 20 | 10.5 (1.4-26.4) | 19 | 27.7 (9.3-39.3) | 20 |
| Cambodia | FARTM | Pailin | 2008-2010 | 6.0 (2.1-10.0) | 5.6 (0.4) | 79 | 8.9 (1.4-26.4) | 76 | 27.7 (3.7-52.6) | 79 |
| Cambodia | PDKJM | Pursat | 2009-2010 | 6.3 (1.7-16.5) | 5.8 (0.4) | 198 | 10.4 (3.1-70.3) | 197 | 22.8 (5.6-84.6) | 198 |
| Cambodia | PDKJM | Ratanakiri | 2010 | 2.7 (1.1-11.5) | 2.9 (0.4) | 52 | 6.8 (3.0-12.2) | 52 | 11.3 (5.4-34.1) | 52 |
| Cambodia | GKRZR | Tasanh | 2008-2009 | 7.0 (1.5-12.2) | 6.1 (0.5) | 67 | 9.0 (1.4-38.0) | 67 | 23.0 (3.8-61.3) | 67 |
| Cambodia | UFYTP | Pailin | 2011-2012 | 6.1 (1.6-9.0) | 5.7 (0.3) | 98 | 9.5 (2.1-22.3) | 94 | 26.4 (3.7-47.4) | 97 |
| Cambodia | UFYTP | Preah Vihear | 2011-2012 | 2.9 (1.2-12.6) | 3.2 (0.6) | 60 | 8.3 (2.0-42.6) | 58 | 16.2 (3.6-59.5) | 59 |
| Cambodia | UFYTP | Pursat | 2011-2012 | 5.6 (1.7-11.8) | 5.2 (0.4) | 119 | 9.6 (2.3-32.1) | 119 | 25.7 (6.4-66.5) | 119 |
| Cambodia | UFYTP | Ratanakiri | 2011-2012 | 2.7 (1.0-5.0) | 2.7 (0.3) | 59 | 7.2 (2.6-26.0) | 55 | 15.5 (5.6-31.6) | 59 |
| DRC | UFYTP | Kinshasa | 2013 | 1.9 (0.7-7.0) | 1.8 (0.5) | 60 | 6.0 (2.0-16.0) | 56 | 11.3 (3.6-21.6) | 60 |
| Gabon | ADXZX | Lambarene | 2005 | 2.8 (0.7-3.7) | 2.3 (0.5) | 28 | 6.0 (1.1-16.2) | 31 | 11.3 (1.9-23.3) | 31 |
| Gabon | ADXZX | Libreville | 2005-2006 | 2.5 (1.7-4.8) | 2.5 (0.2) | 36 | 7.1 (6.0-34.5) | 39 | 17.1 (10.8-43.2) | 39 |
| Ghana | MEFSC | Kintampo | 2007 | 2.8 (1.6-5.4) | 2.9 (0.2) | 112 | 10.1 (5.6-21.7) | 126 | 19.4 (10.1-34.0) | 126 |
| Kenya | MEFSC | Eldoret | 2006-2007 | 2.0 (1.5-10.2) | 2.1 (0.3) | 74 | 7.0 (4.0-166.4) | 98 | 14.5 (7.2-169.3) | 98 |
| Kenya | MEFSC | Kilifi | 2006-2007 | 2.3 (1.5-3.6) | 2.4 (0.2) | 41 | 8.4 (4.5-19.2) | 48 | 15.7 (8.1-28.0) | 48 |
| Kenya | MEFSC | Pinglikani | 2007 | 2.3 (1.5-4.7) | 2.3 (0.3) | 43 | 7.3 (4.2-25.4) | 47 | 14.9 (7.5-31.5) | 47 |
| Laos | TETAJ | Xepon | 2010 | 1.8 (1.1-2.3) | 1.7 (0.2) | 21 | 5.5 (0.7-17.5) | 22 | 11.5 (5.7-20.4) | 22 |
| Laos | UFYTP | Attapeu | 2011-2012 | 1.9 (1.0-7.8) | 2.1 (0.5) | 60 | 7.2 (2.4-23.1) | 55 | 12.9 (5.4-28.8) | 55 |
| Mali | BYMYG | Kenieroba | 2010-2011 | 1.9 (0.7-5.3) | 1.9 (0.3) | 255 | 9.7 (3.0-24.0) | 261 | 15.3 (5.4-29.6) | 260 |
| Mali | SRDFP | Sikasso | 2010-2011 | 2.1 (1.3-4.1) | 2.1 (0.2) | 95 | 11.0 (4.0-15.0) | 98 | 15.2 (7.2-22.7) | 98 |
| Myanmar | UFYTP | Shwe Kyin | 2011-2013 | 3.0 (0.7-8.4) | 3.0 (0.5) | 40 | 7.0 (2.3-22.1) | 39 | 14.6 (4.6-35.1) | 40 |
| Nigeria | MEFSC | Calabar | 2007 | 2.6 (1.5-22.4) | 2.8 (0.5) | 92 | 8.4 (4.0-17.0) | 96 | 15.0 (7.2-23.8) | 96 |
| Nigeria | MEFSC | Enugu | 2006-2007 | 2.5 (1.5-5.4) | 2.5 (0.3) | 117 | 6.1 (3.9-16.5) | 142 | 14.4 (7.1-25.7) | 142 |
| Nigeria | MEFSC | Ibadan | 2007 | 2.2 (1.2-6.2) | 2.1 (0.4) | 33 | 5.0 (3.8-18.2) | 40 | 11.8 (6.8-22.9) | 40 |
| Nigeria | MEFSC | Jos | 2007 | 2.7 (1.3-10.9) | 2.6 (0.4) | 60 | 5.0 (3.7-16.5) | 74 | 10.6 (6.6-34.4) | 74 |
| Nigeria | UFYTP | Ilorin | 2011-2012 | 2.6 (1.4-4.0) | 2.6 (0.3) | 13 | 5.4 (3.8-11.7) | 10 | 10.4 (5.7-23.7) | 12 |
| Tanzania | MEFSC | Bagamoyo | 2007 | 2.2 (1.5-4.0) | 2.2 (0.3) | 24 | 11.0 (4.4-27.2) | 30 | 15.9 (8.0-32.6) | 30 |
| Tanzania | MEFSC | Kiwangwa | 2006-2007 | 2.1 (1.3-9.7) | 2.2 (0.3) | 124 | 6.8 (3.9-38.9) | 190 | 14.6 (7.1-44.9) | 190 |
| Thailand | GHNKU | Bangkok | 1998 | 2.2 (1.1-4.6) | 2.2 (0.3) | 37 | 6.0 (3.0-27.7) | 51 | 14.1 (6.1-28.5) | 54 |
| Thailand | UFYTP | Ranong | 2011-2013 | 6.4 (3.1-13.8) | 5.9 (0.5) | 13 | 8.7 (5.1-16.2) | 12 | 30.6 (11.6-44.2) | 12 |
| Thailand | UFYTP | Srisaket | 2011-2013 | 7.0 (1.6-13.9) | 6.1 (0.5) | 35 | 7.7 (2.1-27.3) | 34 | 27.5 (3.9-60.6) | 32 |
| Thailand | UFYTP | Western border | 2011-2012 | 4.6 (1.4-9.0) | 4.7 (0.4) | 60 | 7.7 (2.0-19.8) | 59 | 19.0 (3.7-47.2) | 60 |
| Thailand | MRGRH | Western border | 2001 | 2.7 (1.1-5.9) | 2.7 (0.3) | 132 | 9.1 (0.7-23.5) | 132 | 15.6 (5.4-38.8) | 134 |
| Thailand | MRGRH | Western border | 2002 | 2.6 (1.0-5.9) | 2.5 (0.3) | 155 | 9.4 (2.5-16.6) | 154 | 15.9 (5.4-25.6) | 155 |
| Thailand | MRGRH | Western border | 2003 | 2.7 (1.1-7.9) | 2.7 (0.3) | 169 | 9.1 (3.0-22.2) | 166 | 15.4 (5.4-26.1) | 168 |
| Thailand | MRGRH | Western border | 2004 | 3.2 (0.8-10.5) | 3.1 (0.5) | 109 | 8.7 (0.0-17.3) | 108 | 14.8 (0.0-23.2) | 109 |
| Thailand | MRGRH | Western border | 2005 | 3.7 (1.5-7.9) | 3.4 (0.4) | 97 | 8.8 (3.0-21.0) | 97 | 15.0 (5.4-39.7) | 97 |
| Thailand | MRGRH | Western border | 2006 | 3.0 (0.8-9.7) | 3.0 (0.5) | 338 | 8.9 (0.9-36.8) | 341 | 15.5 (4.6-53.4) | 342 |
| Thailand | MRGRH | Western border | 2007 | 3.0 (0.6-11.3) | 3.1 (0.5) | 211 | 8.5 (3.0-28.4) | 212 | 15.8 (5.4-59.5) | 213 |
| Thailand | MRGRH | Western border | 2008 | 3.2 (1.2-23.7) | 3.3 (0.4) | 455 | 8.6 (1.0-63.0) | 458 | 16.0 (5.4-65.4) | 457 |
| Thailand | DPZDY | Western border | 2008 | 2.7 (1.1-6.2) | 2.7 (0.5) | 20 | 4.8 (2.1-16.9) | 20 | 11.4 (3.8-28.3) | 20 |
| Thailand | MRGRH | Western border | 2009 | 3.2 (0.9-11.5) | 3.2 (0.5) | 273 | 8.8 (0.8-32.2) | 275 | 16.3 (5.4-57.3) | 275 |
| Thailand | QBPQM | Western border | 2009-2010 | 3.5 (0.9-9.4) | 3.3 (0.6) | 79 | 8.6 (1.2-104.4) | 77 | 16.8 (3.5-116.4) | 77 |
| Thailand | MRGRH | Western border | 2010 | 3.7 (1.5-8.5) | 3.6 (0.4) | 108 | 9.0 (3.0-73.5) | 111 | 16.9 (5.4-77.1) | 111 |
| Thailand | MRGRH | Western border | 2011 | 4.9 (1.2-12.9) | 4.5 (0.5) | 101 | 9.7 (3.1-31.1) | 101 | 17.4 (5.5-54.4) | 101 |
| Vietnam | ATMFH | Binh Phuoc | 2010-2011 | 2.7 (1.0-15.4) | 3.2 (0.8) | 54 | 9.8 (3.0-38.7) | 54 | 16.9 (5.4-83.6) | 55 |
| Vietnam | UFYTP | Binh Phuoc | 2011-2012 | 2.9 (0.7-8.9) | 3.1 (0.7) | 59 | 7.6 (2.0-24.3) | 59 | 16.3 (3.6-49.6) | 59 |
| Vietnam | NKTYE | Phuoc Chien | 2008-2009 | 3.0 (2.0-6.5) | 3.1 (0.3) | 58 | 6.5 (4.8-40.1) | 62 | 11.7 (8.6-52.7) | 62 |
| **AL** ^2^  Benin | EDPJN | Cotonou | 2006-2007 | 3.4 (2.3-5.4) | 3.4 (0.2) | 42 | 7.5 (4.9-39.5) | 72 | 14.0 (8.7-41.6) | 72 |
| Burkina Faso | MEFSC | Bobo-Dioulasso | 2007 | 2.6 (2.5-2.7) | 2.6 (0.0) | 2 | 10.2 (10.0-10.3) | 2 | 18.6 (16.1-21.0) | 2 |
| DRC | UFYTP | Kinshasa | 2013 | 2.2 (1.2-4.6) | 2.1 (0.2) | 58 | 9.0 (2.3-18.8) | 49 | 16.7 (4.6-23.6) | 57 |
| Ghana | MEFSC | Kintampo | 2007 | 3.2 (1.8-7.5) | 3.2 (0.3) | 50 | 15.4 (5.9-28.1) | 61 | 20.8 (10.5-36.8) | 61 |
| Kenya | MEFSC | Eldoret | 2006-2007 | 2.2 (1.6-3.5) | 2.3 (0.2) | 38 | 10.2 (4.2-216.8) | 51 | 15.5 (7.6-309.2) | 51 |
| Kenya | CXJYT | Kilifi | 2002-2003 | 3.6 (2.2-7.1) | 3.7 (0.3) | 77 | 14.0 (3.9-32.5) | 84 | 22.3 (7.1-40.3) | 84 |
| Kenya | EDPJN | Kilifi | 2006-2007 | 3.1 (1.3-5.7) | 3.1 (0.3) | 99 | 10.9 (3.8-20.5) | 109 | 20.9 (6.8-32.2) | 109 |
| Kenya | MEFSC | Kilifi | 2006-2007 | 2.6 (1.4-5.7) | 2.6 (0.3) | 20 | 9.7 (4.6-24.5) | 24 | 16.9 (8.4-28.2) | 24 |
| Kenya | EDPJN | Kisumu | 2007 | 3.3 (1.9-7.2) | 3.4 (0.3) | 38 | 11.7 (5.1-26.3) | 43 | 24.2 (9.2-31.2) | 43 |
| Kenya | MEFSC | Pinglilikani | 2007 | 2.7 (1.7-5.0) | 2.8 (0.3) | 21 | 10.5 (4.4-15.9) | 23 | 17.3 (8.0-26.0) | 23 |
| Mali | EDPJN | Bamako | 2006-2007 | 3.3 (2.2-6.0) | 3.3 (0.2) | 106 | 17.5 (4.9-31.7) | 122 | 24.4 (8.7-37.8) | 122 |
| Mozambique | EDPJN | Manhiça | 2006 | 3.9 (1.4-9.2) | 3.8 (0.5) | 41 | 13.2 (4.0-35.9) | 48 | 19.8 (7.2-39.1) | 48 |
| Nigeria | MEFSC | Calabar | 2007 | 2.6 (1.4-20.5) | 2.8 (0.5) | 48 | 11.0 (4.2-19.4) | 49 | 16.1 (7.5-29.2) | 49 |
| Nigeria | MEFSC | Enugu | 2006-2007 | 2.7 (1.5-7.2) | 2.7 (0.3) | 57 | 9.4 (3.9-18.6) | 73 | 16.0 (7.1-23.8) | 73 |
| Nigeria | MEFSC | Ibadan | 2007 | 2.2 (1.4-4.4) | 2.2 (0.3) | 17 | 9.1 (4.0-19.9) | 19 | 15.3 (7.2-30.3) | 19 |
| Nigeria | MEFSC | Jos | 2007 | 2.4 (1.5-4.3) | 2.5 (0.3) | 30 | 4.6 (3.5-16.4) | 38 | 10.4 (6.4-19.8) | 38 |
| Nigeria | CXJYT | Ibadan | 2002-2003 | 3.0 (1.9-7.9) | 3.0 (0.3) | 49 | 4.4 (2.6-27.4) | 75 | 7.9 (4.8-34.3) | 75 |
| Tanzania | MEFSC | Bagamoyo | 2007 | 2.1 (1.7-3.2) | 2.3 (0.3) | 5 | 10.9 (4.4-72.9) | 13 | 16.5 (7.9-74.2) | 13 |
| Tanzania | EDPJN | Dar es Salaam | 2006-2007 | 3.0 (2.1-7.0) | 3.1 (0.3) | 129 | 13.4 (4.6-53.9) | 173 | 25.0 (8.4-60.6) | 173 |
| Tanzania | CXJYT | Kisarawe | 2002 | 3.0 (2.0-6.0) | 2.9 (0.3) | 62 | 15.3 (3.9-24.4) | 73 | 22.2 (7.0-36.2) | 73 |
| Tanzania | MEFSC | Kiwangwa | 2006-2007 | 2.2 (1.5-5.6) | 2.4 (0.3) | 53 | 12.0 (4.0-26.0) | 94 | 16.7 (7.2-46.0) | 94 |
| Tanzania | MSDDE | Fukayosi | 2006 | 2.7 (0.6-5.6) | 2.5 (0.6) | 43 | 7.8 (1.2-14.9) | 50 | 14.9 (3.0-30.2) | 50 |
| Tanzania | EDPJN | Zanzibar | 2006-2007 | 3.7 (2.3-5.6) | 3.7 (0.2) | 24 | 12.8 (5.3-24.3) | 24 | 23.5 (9.5-31.6) | 24 |
| Uganda | PNUNE | Mbarara | 2005 | 2.8 (1.5-5.1) | 2.7 (0.3) | 41 | 5.4 (3.0-16.4) | 41 | 11.7 (5.4-24.4) | 41 |
| Thailand | EFTTU | Bangkok | 1996-1997 | 3.0 (1.2-6.5) | 3.0 (0.3) | 60 | 11.9 (2.3-38.5) | 62 | 18.7 (4.7-43.1) | 65 |
| Thailand | GHNKU | Bangkok | 1998 | 2.4 (0.9-5.1) | 2.4 (0.4) | 89 | 6.7 (1.1-40.2) | 155 | 14.6 (2.0-46.1) | 161 |
| **DP**^2^  Vietnam | ATMFH | Binh Phuoc | 2010-2011 | 3.0 (1.3-9.1) | 3.3 (0.6) | 55 | 9.3 (3.1-27.7) | 54 | 16.5 (5.6-49.0) | 55 |

^1^Coefficient of variation CV is calculated as sqrt( exp(σ^2^)-1), where σ is the standard deviation after the log transformation.

^2^Treatment groups are defined as: (a) artesunate alone or in combination with a partner drug, daily dose 2 mg/kg (AS 2 mg/kg); (b) artesunate alone or in combination with a partner drug, (c) daily dose 4 mg/kg (AS 4 mg/kg); standard 6-dose regimen of artemether-lumefantrine (AL); (d) standard 3-dose regimen of dihydroartemisinin-piperaquine (DP).
