# Supplementary material for: Baseline data of parasite clearance in patients with falciparum malaria treated with an artemisinin derivative: an individual patient data meta-analysis
Source: Malar J. 2015 Sep 22;14:359. doi: 10.1186/s12936-015-0874-1 (PMC4578675; doi:10.1186/s12936-015-0874-1)
Supplement: Supplementary file 5 — Additional file 5: Table S4. Summary of parasitological measures II: Proportion of profiles with PC1/2 above cut-off of 3, 4, 5 and 6 h presented by study location, year and treatment. [file 12936_2015_874_MOESM5_ESM.docx]

**Table S4 Summary of parasitological measures II: proportion of profiles with PC_1/2_ above cut-off**

|  | Study ID | Study site | Study years | % (n/N) of profiles above cut-off | | | | |
| --- | --- | --- | --- | --- | --- | --- | --- | --- |
|  |  |  |  | 3 hours | 4 hours | 5 hours | | 6 hours |
| **AS 2 mg/kg**^1^ Bangladesh | HUZJF | Bandarban | 2008-2009 | 47 (23/49) | 14 (7/49) | | 2 (1/49) | 2 (1/49) |
| Bangladesh | UFYTP | Ramu | 2012 | 43 (12/28) | 11 (3/28) | | 4 (1/28) | 0 (0/28) |
| Cambodia | PDGZZ | Pailin | 2007 | 95 (19/20) | 95 (19/20) | | 60 (12/20) | 45 (9/20) |
| Cambodia | GKRZR | Tasanh | 2008-2009 | 92 (69/75) | 88 (66/75) | | 79 (59/75) | 67 (50/75) |
| Cambodia | UFYTP | Preah Vihear | 2011-2012 | 57 (34/60) | 27 (16/60) | | 23 (14/60) | 13 (8/60) |
| Cambodia | UFYTP | Ratanakiri | 2011-2012 | 55 (33/60) | 18 (11/60) | | 8 (5/60) | 3 (2/60) |
| Kenya | TZDRS | Pingilikani | 2011 | 30 (47/155) | 3 (5/155) | | 1 (1/155) | 0 (0/155) |
| Kenya | UFYTP | Pingilikani | 2011 | 37 (21/57) | 2 (1/57) | | 0 (0/57) | 0 (0/57) |
| Laos | TETAJ | Xepon | 2010 | 5 (1/22) | 0 (0/22) | | 0 (0/22) | 0 (0/22) |
| Laos | UFYTP | Attapeu | 2011-2012 | 14 (8/58) | 5 (3/58) | | 3 (2/58) | 3 (2/58) |
| Myanmar | UFYTP | Shwe Kyin | 2011-2012 | 57 (23/40) | 30 (12/40) | | 15 (6/40) | 13 (5/40) |
| Nigeria | UFYTP | Ilorin | 2011-2012 | 29 (5/17) | 6 (1/17) | | 6 (1/17) | 6 (1/17) |
| Thailand | DPZDY | Western border | 2008 | 70 (14/20) | 25 (5/20) | | 20 (4/20) | 10 (2/20) |
| Thailand | UFYTP | Western border | 2011-2012 | 83 (48/58) | 78 (45/58) | | 48 (28/58) | 29 (17/58) |
| Thailand | UFYTP | Ranong | 2011-2012 | 80 (8/10) | 70 (7/10) | | 60 (6/10) | 20 (2/10) |
| Vietnam | ATMFH | Binh Phuoc | 2010-2011 | 64 (35/55) | 44 (24/55) | | 38 (21/55) | 25 (14/55) |
| Vietnam | UFYTP | Binh Phuoc | 2011-2012 | 53 (31/59) | 36 (21/59) | | 29 (17/59) | 24 (14/59) |
| **AS 4 mg/kg**^1^ Bangladesh | HUZJF | Bandarban | 2008-2009 | 50 (24/48) | 15 (7/48) | | 4 (2/48) | 0 (0/48) |
| Bangladesh | UFYTP | Ramu | 2012 | 26 (7/27) | 7 (2/27) | | 0 (0/27) | 0 (0/27) |
| Burkina Faso | MEFSC | Bobo-Dioula | 2007 | 33 (1/3) | 0 (0/3) | | 0 (0/3) | 0 (0/3) |
| Cambodia | PDGZZ | Pailin | 2007 | 95 (19/20) | 90 (18/20) | | 75 (15/20) | 45 (9/20) |
| Cambodia | FARTM | Pailin | 2008-2010 | 92 (73/79) | 86 (68/79) | | 68 (54/79) | 52 (41/79) |
| Cambodia | PDKJM | Pursat | 2009-2010 | 94 (187/198) | 85 (169/198) | | 70 (139/198) | 56 (110/198) |
| Cambodia | PDKJM | Ratanakiri | 2010 | 38 (20/52) | 10 (5/52) | | 4 (2/52) | 2 (1/52) |
| Cambodia | GKRZR | Tasanh | 2008-2009 | 91 (61/67) | 84 (56/67) | | 81 (54/67) | 70 (47/67) |
| Cambodia | UFYTP | Pailin | 2011-2012 | 96 (94/98) | 86 (84/98) | | 72 (71/98) | 55 (54/98) |
| Cambodia | UFYTP | Preah Vihear | 2011-2012 | 45 (27/60) | 27 (16/60) | | 20 (12/60) | 17 (10/60) |
| Cambodia | UFYTP | Pursat | 2011-2012 | 89 (106/119) | 78 (93/119) | | 61 (73/119) | 42 (50/119) |
| Cambodia | UFYTP | Ratanakiri | 2011-2012 | 47 (28/59) | 7 (4/59) | | 2 (1/59) | 0 (0/59) |
| DRC | UFYTP | Kinshasa | 2013 | 8 (5/60) | 5 (3/60) | | 3 (2/60) | 3 (2/60) |
| Gabon | ADXZX | Lambarene | 2005 | 43 (12/28) | 0 (0/28) | | 0 (0/28) | 0 (0/28) |
| Gabon | ADXZX | Libreville | 2005-2006 | 11 (4/36) | 3 (1/36) | | 0 (0/36) | 0 (0/36) |
| Ghana | MEFSC | Kintampo | 2007 | 43 (48/112) | 8 (9/112) | | 1 (1/112) | 0 (0/112) |
| Kenya | MEFSC | Eldoret | 2006-2007 | 7 (5/74) | 1 (1/74) | | 1 (1/74) | 1 (1/74) |
| Kenya | MEFSC | Kilifi | 2006-2007 | 27 (11/41) | 0 (0/41) | | 0 (0/41) | 0 (0/41) |
| Kenya | MEFSC | Pinglikani | 2007 | 14 (6/43) | 5 (2/43) | | 0 (0/43) | 0 (0/43) |
| Laos | TETAJ | Xepon | 2010 | 0 (0/21) | 0 (0/21) | | 0 (0/21) | 0 (0/21) |
| Laos | UFYTP | Attapeu | 2011-2012 | 17 (10/60) | 10 (6/60) | | 10 (6/60) | 5 (3/60) |
| Mali | BYMYG | Kenieroba | 2010-2011 | 8 (20/255) | 2 (4/255) | | 1 (2/255) | 0 (0/255) |
| Mali | SRDFP | Sikasso | 2010-2011 | 9 (9/95) | 2 (2/95) | | 0 (0/95) | 0 (0/95) |
| Myanmar | UFYTP | Shwe Kyin | 2011-2013 | 50 (20/40) | 23 (9/40) | | 13 (5/40) | 10 (4/40) |
| Nigeria | MEFSC | Calabar | 2007 | 37 (34/92) | 17 (16/92) | | 11 (10/92) | 5 (5/92) |
| Nigeria | MEFSC | Enugu | 2006-2007 | 24 (28/117) | 5 (6/117) | | 1 (1/117) | 0 (0/117) |
| Nigeria | MEFSC | Ibadan | 2007 | 15 (5/33) | 6 (2/33) | | 3 (1/33) | 3 (1/33) |
| Nigeria | MEFSC | Jos | 2007 | 20 (12/60) | 7 (4/60) | | 3 (2/60) | 3 (2/60) |
| Nigeria | UFYTP | Ilorin | 2011-2012 | 23 (3/13) | 8 (1/13) | | 0 (0/13) | 0 (0/13) |
| Tanzania | MEFSC | Bagamoyo | 2007 | 21 (5/24) | 4 (1/24) | | 0 (0/24) | 0 (0/24) |
| Tanzania | MEFSC | Kiwangwa | 2006-2007 | 13 (16/124) | 5 (6/124) | | 2 (3/124) | 2 (2/124) |
| Thailand | GHNKU | Bangkok | 1998 | 11 (4/37) | 5 (2/37) | | 0 (0/37) | 0 (0/37) |
| Thailand | UFYTP | Western border | 2011-2012 | 82 (49/60) | 63 (38/60) | | 48 (29/60) | 38 (23/60) |
| Thailand | UFYTP | Srisaket | 2011-2013 | 94 (33/35) | 80 (28/35) | | 66 (23/35) | 57 (20/35) |
| Thailand | UFYTP | Ranong | 2011-2013 | 100 (13/13) | 77 (10/13) | | 62 (8/13) | 54 (7/13) |
| Thailand | MRGRH | Western border | 2001 | 36 (48/132) | 8 (11/132) | | 3 (4/132) | 0 (0/132) |
| Thailand | MRGRH | Western border | 2002 | 32 (49/155) | 8 (12/155) | | 1 (2/155) | 0 (0/155) |
| Thailand | MRGRH | Western border | 2003 | 33 (56/169) | 11 (18/169) | | 6 (10/169) | 2 (3/169) |
| Thailand | MRGRH | Western border | 2004 | 60 (65/109) | 27 (29/109) | | 12 (13/109) | 6 (6/109) |
| Thailand | MRGRH | Western border | 2005 | 64 (62/97) | 41 (40/97) | | 16 (16/97) | 6 (6/97) |
| Thailand | MRGRH | Western border | 2006 | 51 (172/338) | 32 (107/338) | | 12 (40/338) | 5 (18/338) |
| Thailand | MRGRH | Western border | 2007 | 50 (106/211) | 29 (62/211) | | 15 (32/211) | 7 (15/211) |
| Thailand | DPZDY | Western border | 2008 | 40 (8/20) | 20 (4/20) | | 10 (2/20) | 5 (1/20) |
| Thailand | MRGRH | Western border | 2008 | 56 (256/455) | 31 (142/455) | | 15 (69/455) | 7 (31/455) |
| Thailand | MRGRH | Western border | 2009 | 54 (147/273) | 33 (90/273) | | 18 (48/273) | 10 (27/273) |
| Thailand | QBPQM | Western border | 2009-2010 | 58 (46/79) | 41 (32/79) | | 24 (19/79) | 16 (13/79) |
| Thailand | MRGRH | Western border | 2010 | 66 (71/108) | 44 (48/108) | | 27 (29/108) | 14 (15/108) |
| Thailand | MRGRH | Western border | 2011 | 79 (80/101) | 62 (63/101) | | 50 (50/101) | 28 (28/101) |
| Vietnam | ATMFH | Binh Phuoc | 2010-2011 | 44 (24/54) | 39 (21/54) | | 35 (19/54) | 28 (15/54) |
| Vietnam | UFYTP | Binh Phuoc | 2011-2012 | 49 (29/59) | 42 (25/59) | | 27 (16/59) | 20 (12/59) |
| Vietnam | NKTYE | Phuoc Chien | 2008-2009 | 50 (29/58) | 14 (8/58) | | 2 (1/58) | 2 (1/58) |
| **AL^1^**  Benin | EDPJN | Benin | 2006-2007 | 69 (29/42) | 21 (9/42) | | 10 (4/42) | 0 (0/42) |
| Burkina Faso | MEFSC | Bobo-Dioula | 2007 | 0 (0/2) | 0 (0/2) | | 0 (0/2) | 0 (0/2) |
| DRC | UFYTP | Kinshasa | 2013 | 2 (1/58) | 2 (1/58) | | 0 (0/58) | 0 (0/58) |
| Ghana | MEFSC | Kintampo | 2007 | 60 (30/50) | 16 (8/50) | | 2 (1/50) | 2 (1/50) |
| Kenya | MEFSC | Eldoret | 2006-2007 | 13 (5/38) | 0 (0/38) | | 0 (0/38) | 0 (0/38) |
| Kenya | CXJYT | Kilifi | 2002-2003 | 73 (56/77) | 35 (27/77) | | 18 (14/77) | 6 (5/77) |
| Kenya | EDPJN | Kilifi | 2006-2007 | 57 (56/99) | 18 (18/99) | | 5 (5/99) | 0 (0/99) |
| Kenya | MEFSC | Kilifi | 2006-2007 | 15 (3/20) | 5 (1/20) | | 5 (1/20) | 0 (0/20) |
| Kenya | EDPJN | Kisumu | 2007 | 74 (28/38) | 24 (9/38) | | 11 (4/38) | 3 (1/38) |
| Kenya | MEFSC | Pinglilikani | 2007 | 29 (6/21) | 14 (3/21) | | 5 (1/21) | 0 (0/21) |
| Mali | EDPJN | Bamako | 2006-2007 | 64 (68/106) | 18 (19/106) | | 6 (6/106) | 1 (1/106) |
| Mozambique | EDPJN | Manhiça | 2006 | 73 (30/41) | 49 (20/41) | | 24 (10/41) | 20 (8/41) |
| Nigeria | MEFSC | Calabar | 2007 | 42 (20/48) | 15 (7/48) | | 8 (4/48) | 2 (1/48) |
| Nigeria | MEFSC | Enugu | 2006-2007 | 35 (20/57) | 12 (7/57) | | 4 (2/57) | 2 (1/57) |
| Nigeria | MEFSC | Ibadan | 2007 | 24 (4/17) | 6 (1/17) | | 0 (0/17) | 0 (0/17) |
| Nigeria | MEFSC | Jos | 2007 | 23 (7/30) | 7 (2/30) | | 0 (0/30) | 0 (0/30) |
| Nigeria | CXJYT | Ibadan | 2002-2003 | 47 (23/49) | 16 (8/49) | | 2 (1/49) | 2 (1/49) |
| Tanzania | MEFSC | Bagamoyo | 2007 | 20 (1/5) | 0 (0/5) | | 0 (0/5) | 0 (0/5) |
| Tanzania | EDPJN | Dar es Salaam | 2006-2007 | 50 (64/129) | 19 (24/129) | | 3 (4/129) | 2 (2/129) |
| Tanzania | CXJYT | Kisarawe | 2002 | 47 (29/62) | 10 (6/62) | | 2 (1/62) | 0 (0/62) |
| Tanzania | MEFSC | Kiwangwa | 2006-2007 | 19 (10/53) | 9 (5/53) | | 4 (2/53) | 0 (0/53) |
| Tanzania | MSDDE | Fukayosi | 2006 | 42 (18/43) | 14 (6/43) | | 5 (2/43) | 0 (0/43) |
| Tanzania | EDPJN | Zanzibar | 2006-2007 | 83 (20/24) | 33 (8/24) | | 13 (3/24) | 0 (0/24) |
| Thailand | EFTTU | Bangkok | 1996-1997 | 50 (30/60) | 20 (12/60) | | 7 (4/60) | 2 (1/60) |
| Thailand | GHNKU | Bangkok | 1998 | 28 (25/89) | 6 (5/89) | | 1 (1/89) | 0 (0/89) |
| Uganda | PNUNE | Mbarara | 2005 | 37 (15/41) | 7 (3/41) | | 2 (1/41) | 0 (0/41) |
| **DP**^1^  Vietnam | ATMFH | Binh Phuoc | 2010-2011 | 47 (26/55) | 36 (20/55) | | 33 (18/55) | 18 (10/55) |

^1^Treatment groups are defined as: (a) artesunate alone or in combination with a partner drug, daily dose 2 mg/kg (AS 2 mg/kg); (b) artesunate alone or in combination with a partner drug, daily dose 4mg/kg (AS 4mg/kg); (c) standard 6-dose regimen of artemether-lumefantrine (AL); (d) standard 3-dose regimen of dihydroartemisinin-piperaquine (DP).
